# Sugar consumption and its role in dental caries: Insights from a 2-sample Mendelian randomization study

**DOI:** 10.1097/MD.0000000000046479

**Published:** 2025-12-19

**Authors:** Sun Woo Lim, Junhua Wu, Zhihuan Tian, Yeon Woo Kim, Seongjin Lim, Seung Gyu Choi, Hyewon Park, Joon Won Kang, Jin-Young Choi, Dong Woon Kim

**Affiliations:** a Department of Oral Anatomy and Neurobiology, Kyung Hee University College of Dentistry, Seoul, Republic of Korea; b Department of Neurology, Southwest Hospital, Third Military Medical University (Army Medical University), Chongqing, China; c Department of Medical Science, Chungnam National University College of Medicine, Daejeon, Republic of Korea; d Department of Pediatrics, Chungnam National University Hospital, Daejeon, Republic of Korea; e Department of Orthodontics, Kyung Hee University College of Dentistry, Kyung Hee University Medical Center, Seoul, Republic of Korea.

**Keywords:** dental caries, Mendelian randomnization, metabolic disorder, SNPs, sugar consumption

## Abstract

Dental caries is a prevalent chronic disease influenced by both genetic and environmental factors, particularly dietary. Using a 2-sample Mendelian randomization (MR) approach with genome-wide association study data, this study examined the potential causal relationship between sugar preference, diabetes, and dental caries. The research seeks to clarify the influence of genetic predispositions and metabolic conditions, such as diabetes, on dental health. We conducted a 2-sample MR analysis using genome-wide significant single nucleotide polymorphisms (*P* < 5 × 10⁻⁸), linkage disequilibrium pruning (*R*^2^ < 0.001), and multiple MR methods including inverse-variance weighted, MR-Egger, robust adjusted profile score, and MR-PRESSO. Genetic variants associated with exposures were harmonized with outcome data using the 2 sample MR R package, and ambiguous palindromic single nucleotide polymorphisms were excluded. We found no strong evidence for a causal effect of genetically predicted sugar intake on dental caries. However, both type 1 and type 2 diabetes showed positive associations with caries experience, suggesting that metabolic conditions may contribute to caries risk independently of sugar intake. No significant genetic correlation was observed between sugar preference and diabetes, and Steiger filtering supported the directionality of our analysis. Findings from sensitivity analyses (e.g., leave-one-out, MR-PRESSO) confirmed the robustness of the results. Our findings suggest that the traditionally assumed causal role of sugar intake in dental caries may be overstated. Instead, they support a model in which genetic predispositions and metabolic disorders such as diabetes may play a more substantial role. These findings advocate for personalized dental care approaches that consider individual genetic predispositions and metabolic conditions, offering new insights for future research and clinical practice.

## 1. Introduction

Dental caries, commonly referred to as tooth decay, is one of the most widespread chronic diseases globally.^[1]^ It impacts individuals across all age groups due to a complex interaction between host factors – such as saliva composition and tooth enamel integrity – and environmental influences, particularly diet and oral hygiene. Caries begins with the demineralization of tooth enamel, caused by acids produced when bacteria ferment dietary sugars.^[2,3]^

Traditionally, poor oral hygiene and a diet high in cariogenic foods – those rich in fermentable sugars, including chocolates and sugar-sweetened beverages – have been recognized as significant risk factors for dental caries.^[4,5]^ Numerous studies have consistently demonstrated a strong association between sugar intake and the incidence of dental caries.^[6,7]^ However, the development of caries is not uniform among individuals with similar dietary patterns. Not all individuals with high sugar consumption develop caries, raising questions about underlying factors beyond sugar intake alone.

Recent research has increasingly highlighted the role of genetics in the risk of dental caries, showing that both genetic predispositions and environmental factors contribute to caries development.^[8,9]^ Specifically, genetic variations that affect saliva composition, enamel structure, and immune response have been implicated in caries susceptibility. Furthermore, genetic differences that influence taste perception and dietary preferences, particularly for sweet foods, may also impact the risk of developing dental caries. For example, genetic variations in the TAS1R3 and GNAT3 genes are associated with heightened sensitivity to sweet tastes, potentially leading to increased sugar consumption and, consequently, a higher risk of caries.^[10,11]^

The American Dental Association classifies dental caries into 3 stages: initial lesion, moderate lesion, and advanced lesion, which correspond to caries affecting the enamel, dentin, and pulp, respectively.^[2,12]^ This classification system aids in the diagnosis and selection of appropriate treatment strategies based on disease severity. For instance, initial lesions may be managed with noninvasive treatments, whereas advanced lesions often require more complex interventions, such as root canal therapy.

While the association between sugar consumption and dental caries is well-documented, the causal relationship remains uncertain due to potential confounding and reverse causality inherent in observational studies. Moreover, most dietary assessments rely on self-reported sugar intake, which is prone to measurement error and recall bias.

We selected sugar-related exposures (e.g., chocolate intake, sugar-added foods, sugar-sweetened beverages, fruit intake) and relevant metabolic outcomes (e.g., dental caries, type 1 and type 2 diabetes, HOMA-IR, HOMA-B) based on prior epidemiologic evidence linking these factors with dental caries risk. These traits were chosen to capture different sources and patterns of sugar consumption that may influence oral health through distinct biological and behavioral pathways.

To minimize the potential for collider bias, we used ancestry-matched exposure and outcome datasets and excluded single nucleotide polymorphisms (SNPs) associated with potential confounders or outcomes using LDlink and genome-wide association study (GWAS) catalog searches.

Additionally, the relationship between diabetes – a condition closely linked to high sugar intake – and dental caries is of particular interest.^[13]^ Type 2 diabetes, in particular, is associated with various lifestyle factors, including diet, and has been shown to increase the risk of both periodontal disease and dental caries.^[14,15]^

To overcome these limitations and disentangle correlation from causation, we apply a 2-sample Mendelian randomization (MR) approach using large-scale GWAS data. MR analysis utilizes genetic variants as instrumental variables (IVs) to infer causality, thereby mitigating biases arising from confounding factors and reverse causation. This method is particularly effective in distinguishing correlation from causation in observational data.^[16,17]^

We hypothesize that genetic predispositions to prefer sweet foods may increase the risk of dental caries, but this risk is likely moderated by other factors, including the presence of diabetes. To explore this hypothesis, we also examine the interaction between diabetes and sugar consumption in the development of dental caries. Recognizing that not all individuals with high sugar intake develop caries, we focus on the interplay between genetic liability, dietary behaviors, and metabolic conditions such as diabetes.

This study aims to investigate the causal relationships between sugar consumption, genetic predispositions, and dental caries experience through a 2-sample MR approach. Additionally, we examine how type 1 and type 2 diabetes influence this relationship using bidirectional and sensitivity MR analyses. By leveraging genetic instruments and addressing population stratification, pleiotropy, and measurement error, this study seeks to provide more robust evidence regarding the etiology of dental caries. Furthermore, this study highlights the potential for future research to explore the mechanisms by which genetic factors and lifestyle choices interact to influence oral health outcomes.

## 2. Methods

### 2.1. Study design

This study aimed to evaluate the causal relationship between specific exposure variables and outcomes using MR analysis. We utilized summary statistics derived from GWAS. The key to MR analysis is the appropriate selection of IVs, which in this study were relevant and independent SNPs. To improve causal inference, we applied multiple MR methods, addressed potential pleiotropy, and validated assumptions through sensitivity analyses such as MR-Egger, robust adjusted profile score (RAPS), and MR-PRESSO.^[17,18]^

### 2.2. Instrumental variable selection

In this study, we carefully selected SNPs to serve as IVs for our MR analysis. The goal was to ensure that the SNPs used were both relevant to the exposure of interest and free from confounding factors that could bias the results.

### 2.3. Initial SNP selection

To ensure independence and minimize complications arising from linkage disequilibrium (LD), we applied LD pruning with an *R*² threshold <0.001. This prevents inflated statistical power or multicollinearity. The initial significance threshold was set at *P* < 1 × 10⁻⁵, and further refined to *P* < 5 × 10⁻⁸ when the number of SNPs exceeded 100, ensuring strong instruments. We initially applied a relaxed threshold (*P* < 1 × 10⁻⁵) to identify a sufficiently large set of candidate SNPs, which is consistent with previous MR studies when the number of genome-wide significant SNPs is limited. This approach balances the risk of false negatives (by not excluding potentially valid instruments) with the risk of false positives, which we further minimized through LD pruning (*R*² < 0.001), harmonization, and multiple sensitivity analyses. When more than 100 SNPs passed the initial threshold, we further refined the selection to *P* < 5 × 10⁻⁸ to ensure robust instrument strength. This cutoff was chosen to prioritize stronger genetic instruments and reduce the risk of weak instrument bias, while ensuring a manageable number of SNPs for analysis and interpretation. This approach is consistent with previous MR studies where a large number of initial instruments could compromise statistical validity and computational feasibility. LD refers to the nonrandom co-inheritance of nearby alleles, which can introduce several methodological challenges:

1. Biased results: when SNPs are closely linked, it becomes challenging to determine which SNP is actually causing an effect. This can result in 1 SNP’s effect being mistaken for another’s, leading to inaccurate research conclusions.^[19]^
2. Reduced statistical power: using SNPs that are correlated with each other decreases the amount of independent information available, reducing the study’s ability to detect true genetic associations. It’s like hearing the same information repeatedly without gaining new insights.^[20]^
3. Multicollinearity issues: if multiple SNPs are too similar in behavior, the statistical analysis can become unstable, making it difficult to draw accurate conclusions. This is akin to a puzzle where too many pieces look alike, complicating the process of finding the correct fit.^[21]^
4. Increased complexity in interpretation: closely linked SNPs make it difficult to determine which one is actually exerting an effect, leading to challenges in distinguishing the SNP that is truly important from one that is merely associated by proximity.

To avoid these issues, we set a strict threshold of *R*^2^ < 0.001 for SNP selection. The *R*^2^ value measures the degree of correlation between 2 SNPs, with a value of 1 indicating per^[17]^ year selecting SNPs with *R*^2^ < 0.001, we ensured that the SNPs included in our analysis were largely independent, thus maintaining the statistical validity of the MR analysis. The initial selection criterion for SNPs was based on their association with the exposure variable, with a significance level set at *P* < 1 × 10^−5^.^[17,18]^

SNPs were selected as IVs based on genome-wide significance (*P* < 5 × 10⁻⁸) for the exposure variable to satisfy the relevance criterion. LD pruning (*R*² < 0.001) was applied to ensure independence between SNPs. We used LDlink to exclude SNPs previously reported to be associated with potential confounders or outcomes (e.g., dental caries, periodontitis) and to remove proxy SNPs with indirect trait associations. Where the lead SNP was not available in the outcome dataset, we identified a proxy SNP with *R*² > 0.8. All SNPs were harmonized between exposure and outcome datasets, and sensitivity analyses (MR-Egger, weighted median, and RAPS) were used to evaluate potential violations of the IV assumptions. LD between SNPs was assessed using the *R*² metric (European 1000 genomes reference panel) to ensure independence among SNPs. Additionally, LDlink was used to exclude SNPs indirectly associated with potential confounders or related traits, further minimizing hidden correlations between variants.

### 2.4. Adjusting for multiple SNPs

When the initial selection process resulted in a large number of SNPs (>100), a more stringent significance threshold of *P* < 5 × 10^−8^ was applied to refine the selection. This approach ensured that only the most strongly associated SNPs were included. However, if the number of SNPs still exceeded 100 even after applying this stricter criterion, we retained all selected SNPs for the analysis. This method allowed us to balance the need for robust and reliable genetic instruments with the practical limitations of conducting the analysis.^[17,22]^

### 2.5. Ensuring validity of instrumental variables

For SNPs to be valid IVs in MR analysis, they must satisfy 3 key assumptions:

1. Relevance: the selected SNPs must have a strong association with the exposure variable, ensuring that the SNPs are good predictors of the exposure, which is crucial for detecting a causal relationship with the outcome.^[17,22]^
2. Independence: the SNPs must not be associated with any confounding variables that could affect both the exposure and the outcome. This ensures that any observed association between the SNPs and the outcome is due to the exposure and not to some other factor.^[18,23]^
3. Exclusivity: the SNPs must influence the outcome only through their effect on the exposure, and not through any other pathways (no horizontal pleiotropy).^[23,24]^

To verify these assumptions, we used the online platform LDlink (https://ldlink.nci.nih.gov/, accessed on August 12, 2024) to screen and eliminate SNPs that were linked to potential confounders or outcomes such as dental caries and periodontitis. This process also involved removing proxy SNPs, which are SNPs that might indirectly influence the outcome through their association with other traits (dental caries, periodontitis, or other oral phenotypes based on previous GWAS data).^[22,23]^

### 2.6. Mendelian randomization analysis

For the MR analysis, we utilized the TwoSampleMR R package (version 0.5.7; Hemani et al, https://mrcieu.github.io/TwoSampleMR/) and the MendelianRandomization R package (version 0.6.0) implemented in R version 4.3.2. Palindromic SNPs with intermediate allele frequencies were removed, and Steiger filtering was applied to confirm directionality.

We primarily employed the inverse-variance weighted (IVW) method, which is most efficient when all SNPs are valid instruments. To minimize weak instrument bias, we selected only genome-wide significant SNPs (*P* < 5 × 10⁻⁸) and confirmed that *F*-statistics for each instrument exceeded 10. We also complemented IVW with pleiotropy-robust methods (MR-Egger, weighted median, RAPS) to cross-validate findings. While the weighted median estimator is more robust to invalid instruments, it is less efficient than IVW when most SNPs are valid, which justified using IVW as the primary method.

We additionally applied IVW with multiplicative random effects (IVW-MRE) to account for heterogeneity across SNPs and minimize bias from potential pleiotropy. The weighted median estimator can provide consistent estimates even if up to 50% of the instruments are invalid, while RAPS further adjusts for residual pleiotropy and weak instrument bias. To reduce the risk of false positives arising from multiple testing, we applied false discovery rate correction across all exposures and outcomes. This combined analytical approach minimizes the likelihood that our findings are driven by model misspecification, pleiotropy, or chance findings.

We minimized residual confounding, reverse causality, and verified the 3 core IV assumptions (relevance, independence, exclusivity) by:

1. removing SNPs linked to confounders or outcomes using LDlink and GWAS catalogs,
2. confirming instrument strength with *F*-statistics > 10,
3. applying Steiger filtering, heterogeneity tests (Cochran’s *Q*), MR-Egger intercept test (*P* < .05), and leave-one-out analyses, and
4. excluding SNPs previously associated with potential confounders such as socioeconomic status, oral hygiene, diet quality, and physical activity using LDlink and the GWAS Catalog to further reduce residual confounding.

These procedures found no evidence of substantial horizontal pleiotropy, supporting the validity of our instruments. Together, these stringent SNP selection criteria and pleiotropy-robust sensitivity analyses ensured that the IVs were valid and relevant to the sugar consumption phenotypes. We further minimized potential population stratification and unmeasured confounding by using ancestry-matched exposure and outcome datasets and excluding SNPs associated with confounders or outcomes through LDlink and GWAS catalog searches. Each GWAS used in the analysis had already adjusted for age, sex, genotyping array, and principal components of ancestry, further reducing the risk of bias.

Smoking status was analyzed as a positive control outcome to evaluate instrument validity. Details of the GWAS dataset for smoking status (GWAS ID: ebi-a-GCST90029014; n = 4,68,170; adjusted for age, sex, assessment center, genotyping array, and 10 principal components) are provided in Section 2.10.

### 2.7. Primary method: inverse-variance weighted (IVW)

The primary method we employed was the IVW approach. The IVW method is suitable for situations where multiple IVs are used, as it provides a comprehensive estimate. This is often necessary in practical research because a single IV may not provide robust conclusions. This method combines the estimates of the causal effect from each SNP, weighting them by the inverse of their variance, to provide an overall estimate of the causal effect. The IVW method assumes that all SNPs are valid instruments and that there is no pleiotropy (where a SNP influences the outcome through pathways other than the exposure). When these assumptions hold, IVW provides a precise and unbiased estimate of the causal effect.^[17,23]^ The IVW method is highly dependent on assumptions, such as the need for IVs to be significantly correlated with the exposure and for IVs not to have direct associations with confounding factors. Any violation of these assumptions may affect the validity of the results.

### 2.8. Supplementary methods

Recognizing the possibility of pleiotropy, which could bias the IVW estimates, we employed several supplementary methods:

1. Inverse variance weighted (multiplicative random effects): The IVW method with multiplicative random effects was employed to estimate the causal relationship between the exposure and outcome. This approach combines the individual estimates of the causal effect from each SNP, weighting them by the inverse of their variance. The multiplicative random effects model allows for the presence of heterogeneity across SNPs, accommodating potential differences in the causal estimates due to pleiotropy or other biases. This method adjusts for the variance in effect sizes, improving the accuracy and reliability of causal inference.^[17,24]^
2. Weighted median estimator: The weighted median method provides a robust estimate of the causal effect even if up to 50% of the SNPs are invalid instruments. This method is particularly useful when there is concern about pleiotropy affecting the results.^[23,24]^
3. MR-Egger regression: MR-Egger regression is used to detect and adjust for pleiotropy by allowing the intercept to differ from 0. This method can indicate whether pleiotropy is present andprovides a less biased estimate of the causal effect, though it typically has lower precision compared to IVW.^[23,24]^
4. RAPS: RAPS was applied to further refine the estimates and adjust for any residual pleiotropy, particularly when dealing with weak instruments. This method enhances the robustness of our causal estimates in the presence of pleiotropy.^[25,26]^

### 2.9. Positive control outcome

To establish a positive control outcome, we utilized genetic associations with smoking status, obtained from the MRC-IEU GWAS Database (ID: ebi-a-GCST90029014), which involved up to 4,68,170 White British individuals. By using smoking status as a positive control, we aimed to validate the reliability of our MR analysis methods, as the causal relationship between smoking and various health outcomes is well-established. This approach allowed us to ensure that the IVs and methodologies employed in our analysis were functioning correctly and producing expected results, thereby enhancing the credibility of our findings.

### 2.10. Data sources

All data utilized in this study were obtained from public databases that have undergone formal ethical review and are openly accessible. The study utilized various datasets, each pertaining to specific dietary intakes and health outcomes, particularly focusing on chocolate consumption, sugar-added foods, artificial sweeteners, fruit intake, and diabetes-related measures. A complete description of each GWAS dataset, including trait name, GWAS ID, sample size, population ancestry, cohort source, and genomic coverage, is provided in Table S1, Supplemental Digital Content, https://links.lww.com/MD/Q893. To ensure data integrity, we harmonized all datasets using the *TwoSampleMR* R package and applied standard quality control procedures. SNPs with minor allele frequency < 0.01 or imputation INFO < 0.9 were excluded, and LD pruning (*R*² < 0.001) was conducted to ensure independence among instruments. We further excluded SNPs associated with confounders or outcomes using LDlink and GWAS catalog searches. All datasets included participants of primarily European ancestry, and exposure and outcome GWAS were ancestry-matched to minimize population stratification. Each original GWAS adjusted for age, sex, genotyping array, and at least 10 genetic principal components to control for population stratification and potential confounding. In addition, by using ancestry-matched exposure and outcome GWAS and removing SNPs associated with confounders or outcomes, we minimized the potential for collider bias in our MR analyses.

#### 2.10.1. Chocolate intake

Genetic associations for various chocolate intakes were analyzed using data from the MRC-IEU GWAS Database. The study included approximately 64,945 White British participants from the UK Biobank, adjusting for age, sex, and other factors. Specifically, data were collected for chocolate bar (ID: ukb-b-117), chocolate biscuits (ID: ukb-b-9886), chocolate-covered biscuits (ID: ukb-b-5068), dark chocolate (ID: ukb-b-16139), hot chocolate (ID: ukb-b-16449), milk chocolate (ID: ukb-b-4569), chocolate-covered raisins (ID: ukb-b-1160), chocolate sweets (ID: ukb-b-9835).

#### 2.10.2. Sugar-added food intake

Genetic associations with inverse-normally transformed intakes of cake (ukb-b-3433), doughnut (ukb-b-6214), fizzy drink (ukb-b-2832), fruitcake (ukb-b-17775), ice-cream (ukb-b-17189), pancake (ukb-b-6500), and sweets (ukb-b-10366, ukb-b-10217) were obtained from the MRC-IEU GWAS Database. The study population consisted of 64,949 White British individuals from the UK Biobank.

#### 2.10.3. Practice of adding sugar to food

Genetic associations with sugar added to cereal were obtained from the MRC-IEU GWAS Database (ID: ukb-b-11697), coffee (ID: ukb-b-243), and tea (ID: ukb-b-8442), based on a study population of 64,949 individuals.

#### 2.10.4. Practice of adding artificial sweeteners

Genetic associations with the intake of artificial sweeteners added to cereal (ID: ukb-b-3143), coffee (ID: ukb-b-1338), tea (ID: ukb-b-5867), and low-calorie drinks (ID: ukb-b-19703) were retrieved from the MRC-IEU GWAS Database, with data involving up to 64,949 White British individuals from the UK Biobank.

#### 2.10.5. Intake of fruits containing presumably high sucrose levels

Fruit liking: genetic data for fruit liking were obtained from the GWAS Catalog (ID: GCST90094766), with data collected in 2022.

Fruits intakes: associations for intakes of apple (ukb-b-4070), banana (ukb-b-5362), cherry (ukb-b-2221), mango (ukb-b-19862), melon (ukb-b-6218), peach/nectarine (ukb-b-6154), and pineapple (ukb-b-1164) were sourced from the MRC-IEU GWAS Database, involving up to 64,949 individuals.

#### 2.10.6. Diagnostic criteria for diabetes mellitus

Blood glucose levels: genetic associations with blood glucose levels were sourced from the MRC-IEU GWAS Database (ID: ebi-a-GCST90025986), involving a sample size of up to 4,00,458 individuals. HOMA-B and HOMA-IR: Data for HOMA-B (ID: ieu-b-117) and HOMA-IR (ID: ieu-b-118) were obtained from the MAGIC consortium, with both datasets including up to 36,466 participants from multiple cohorts. Type 1 and type 2 diabetes: genetic data for type 1 diabetes (ID: ebi-a-GCST90014023) were sourced from the University of California, San Diego, and Ulm University, while type 2 diabetes data (ID: ebi-a-GCST90014023) were provided by BioBank Japan, UK Biobank, and FinnGen, involving up to 5,20,580 and 4,90,089 participants, respectively.

### 2.11. Positive control outcome

Smoking status: genetic associations with smoking status were obtained from the MRC-IEU GWAS Database (ID: ebi-a-GCST90029014), involving up to 4,68,170 White British individuals.

### 2.12. Outcome: dental caries

Genetic associations with dental caries were classified into 3 categories based on the criteria specified by the American Dental Association: enamel caries, dentin caries, and pulp caries. These associations were obtained from summary statistics from a meta-analysis available at FinnGen (ID: finngen_R11_K11_CARIES_1_OPER_ONLYAVO, finngen_R11_K11_CARIES_2_OPER_ONLYAVO, finngen_R11_K11_CARIES_3_OPER_ONLYAVO), which can be accessed at (https://www.finngen.fi/en). The study included a well-phenotyped cohort of up to 5,00,000 Finnish individuals.

### 2.13. Outcome: T1DM, T2DM

These associations were obtained from summary statistics from a meta-analysis available at FinnGen (ID: summary_stats_finngen_R11_T1D, summary_stats_finngen_R11_T2D), which can be accessed at (https://www.finngen.fi/en). The study included a well-phenotyped cohort of up to 5,00,000 Finnish individuals.

### 2.14. Ethical approval and consent to participate

Ethical approval was secured for all original studies. The OpenGWAS Database is an open-access dataset, and the GWAS on oral diseases adhered to all pertinent ethical guidelines, including the Declaration of Helsinki. Ethical approval for data collection and analysis was obtained by each study through their respective local ethics committees. In the original studies, all participants gave written informed consent. Our study only used publicly available summary data and did not require additional IRB approval.

## 3. Results

### 3.1. Smoking and dental caries

**Figure 1.**
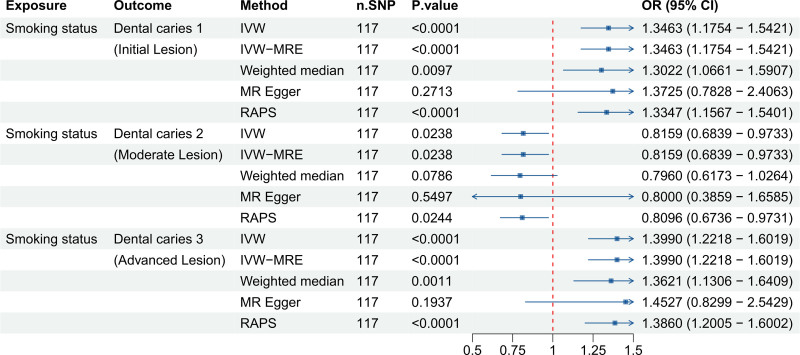
Forest plot illustrating the odds ratios (ORs) and 95% confidence intervals (CIs) for the association between smoking status and the risk of dental caries across 3 stages (dental caries 1, 2, and 3) using different Mendelian randomization (MR) methods (IVW, IVW-MRE, weighted median, MR-Egger, RAPS). Significant associations are highlighted with corresponding ORs and CIs. CI = confidence interval, IVW = inverse-variance weighted, MR = Mendelian randomization, OR = odds ratio, SNP = single nucleotide polymorphism.

To validate our methodological approach, we used smoking status as a positive control because its negative impact on oral health is well-documented (Fig. 1). This outcome was selected to verify the MR framework in line with established causal relationships.

1. Dental caries 1: The IVW analysis revealed a significant positive association between smoking status and Dental caries 1 (*P = *1.77E−05 < .05, OR = 1.3463, OR 95% CI = 1.1754–1.5421). Additionally, sensitivity analysis, including IVW-MRE (*P = *1.77E−05 < .05, OR = 1.3463, OR 95% CI = 1.1754–1.5421), weighted median (*P = *.0097 < .05, OR = 1.3022, OR 95% CI = 1.0661–1.5907), and RAPS (*P = *7.72E−05 < .05, OR = 1.3347, OR 95% CI = 1.1567–1.5401), also showed significant results, indicating a significantly strong positive association.
2. Dental caries 2: The IVW analysis demonstrated a significant negative association between smoking status and Dental Caries 2 (*P = *.0238 < .05, OR = 0.8159, OR 95% CI = 0.6839–0.9733). The RAPS method also indicated significance (*P = *.0244 < .05, OR = 0.8096, OR 95% CI = 0.6736–0.9731), suggesting a significant negative association.
3. Dental caries 3: The IVW analysis indicated a significant positive association between smoking status and dental caries 3 (*P = *1.18E−06 < .05, OR = 1.3990, OR 95% CI = 1.2218–1.6019). The RAPS method supported this finding (*P = *8.50E−06 < .05, OR = 1.3860, OR 95% CI = 1.2005–1.6002), indicating a significantly strong positive association.

These findings validate our IV assumptions and demonstrate the robustness of our MR approach across methods including IVW, IVW-MRE, weighted median, and RAPS.

### 3.2. Chocolate foods and dental caries

**Figure 2.**
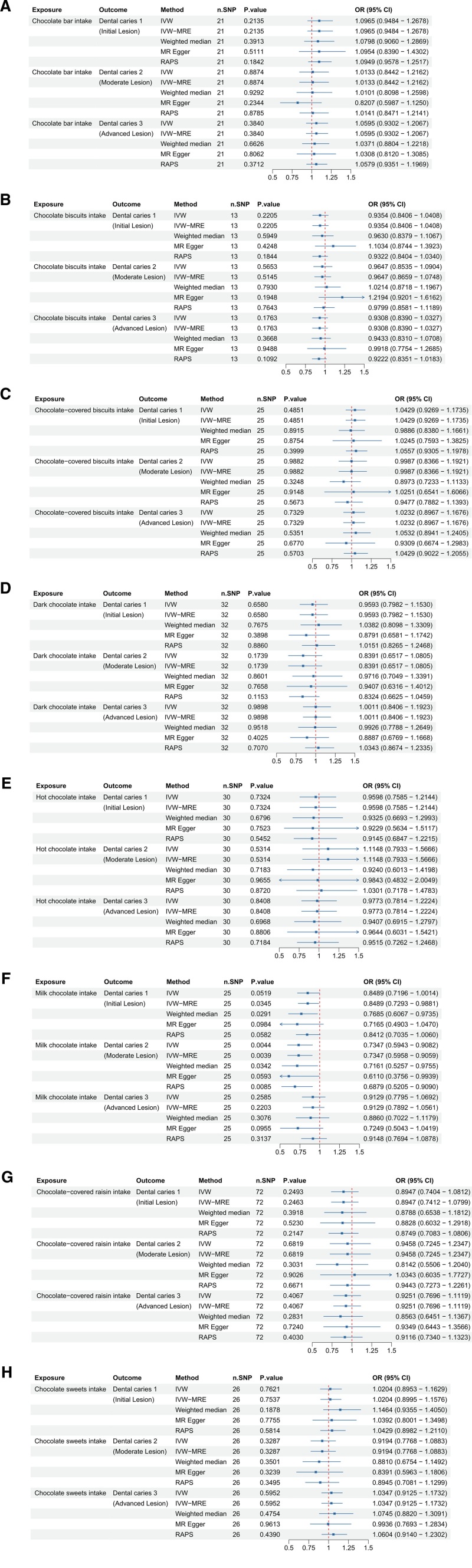
Forest plots illustrating the odds ratios (ORs) and 95% confidence intervals (CIs) for various chocolate intake types and dental caries risk using different Mendelian randomization (MR) methods. (A) Chocolate bar intake; (B) chocolate biscuits intake; (C) chocolate sweet intake; (D) chocolate-covered biscuits intake; (E) chocolate-covered raisin intake; (F) chocolate sweet intake; (G) milk chocolate intake; and (H) hot chocolate intake. Significant findings and the robustness of associations are highlighted across methods such as IVW, IVW-MRE, weighted median, MR-Egger, and RAPS. CI = confidence interval, IVW = inverse-variance weighted, MR = Mendelian randomization, OR = odds ratio, RAPS = robust adjusted profile score, SNP = single nucleotide polymorphism.

To verify the common belief that high sugar intake is a major risk factor for dental caries, we investigated the causal relationship between the consumption of sugar-rich foods – such as chocolate (Fig. 2) – and the risk of developing dental caries (Fig. 2). Initially, we analyzed the association between chocolate bar intake (ID: ukb-b-117) and dental caries outcomes. Dental caries 1: the IVW analysis did not find a significant association between chocolate bar intake and dental caries 1 (*P* = .2135 > .05, OR = 1.0965, OR 95% CI = 0.9484–1.2678). Dental caries 2: no significant association was found between chocolate bar intake and dental caries 2 in the IVW analysis (*P* = .8874 > .05, OR = 1.0133, OR 95% CI = 0.8442–1.2162). Dental caries 3: the IVW analysis did not show a significant association between chocolate bar intake and dental caries 3 (*P* = .3840 > .05, OR = 1.0595, OR 95% CI = 0.9302–1.2067).

Additionally, the investigation included chocolate biscuits (ID: ukb-b-9886), chocolate-covered biscuits (ID: ukb-b-5068), dark chocolate (ID: ukb-b-16139), hot chocolate (ID: ukb-b-16449), milk chocolate (ID: ukb-b-4569), chocolate-covered raisins (ID: ukb-b-1160), and chocolate sweets (ID: ukb-b-9835) with all showing non-significant results for dental caries.

However, milk chocolate demonstrated a different result. For dental caries 1, the association was borderline significant, with additional methods confirming a negative association, suggesting a potential protective effect. Notably, for dental caries 2, a significant negative association was observed, indicating a strong protective effect, supported by multiple analytical methods. No significant association was found for dental caries 3 with milk chocolate. These findings suggest that challenge the general assumption that all sugar-rich foods equally contribute to dental caries, suggesting that some subtypes (e.g., milk chocolate) may be inversely associated with caries risk.

### 3.3. Sugar rich foods and dental caries

**Figure 3.**
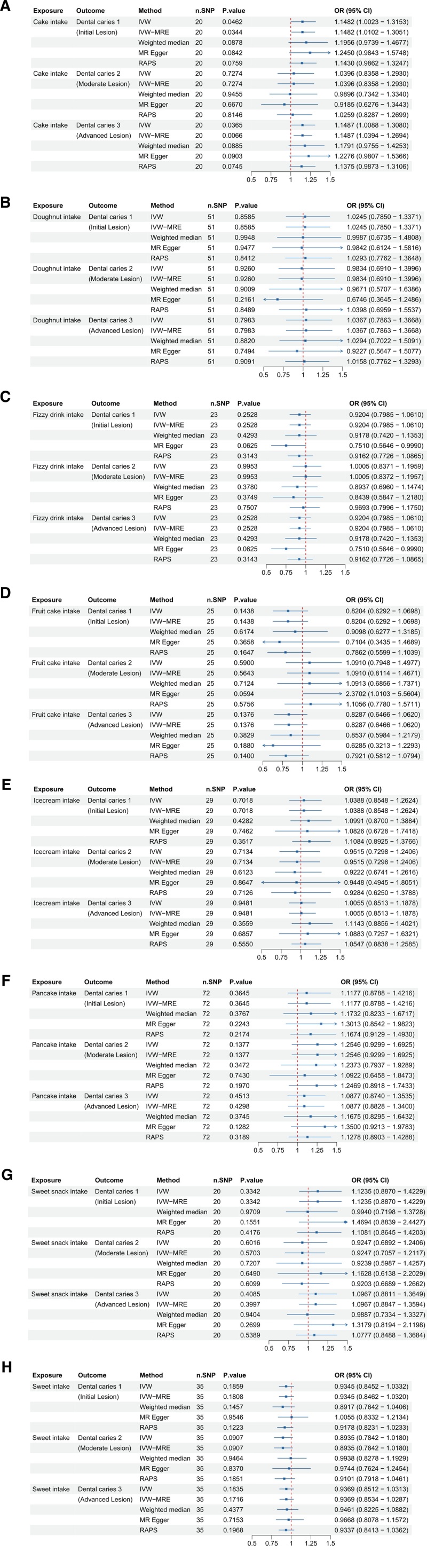
Forest plots illustrating the odds ratios (ORs) and 95% confidence intervals (CIs) for various snack and dessert intake types and dental caries risk using different Mendelian randomization (MR) methods. (A) Cake intake; (B) doughnut intake; (C) fizzy drink intake; (D) fruitcake intake; (E) ice-cream intake; (F) pancake intake; (G) sweet snack intake; and (H) sweets intake. Significant findings and the robustness of these associations are highlighted across methods such as IVW, IVW-MRE, weighted median, MR-Egger, and RAPS. CI = confidence interval, IVW = inverse-variance weighted, MR = Mendelian randomization, OR = odds ratio, RAPS = robust adjusted profile score, SNP = single nucleotide polymorphism.

The study explored the causal relationship between sugar-rich foods and dental caries development (Fig. 3). For cake intake (ukb-b-3433), significant positive associations were found with dental caries 1 and 3, supported by the IVW and IVW-MRE methods, while no significant association was observed for dental caries 2. Doughnut (ukb-b-6214), fizzy drink (ukb-b-2832), fruitcake (ukb-b-17775), ice-cream (ukb-b-17189), pancake (ukb-b-6500), and sweets (ukb-b-10366, ukb-b-10217) showed no significant associations with any dental caries outcomes, as indicated by *P*-values > .05. These findings demonstrate the heterogeneity of dietary sugar sources and their differential impact on dental outcomes.

### 3.4. The practice of adding sugar to food and dental caries

**Figure 4.**
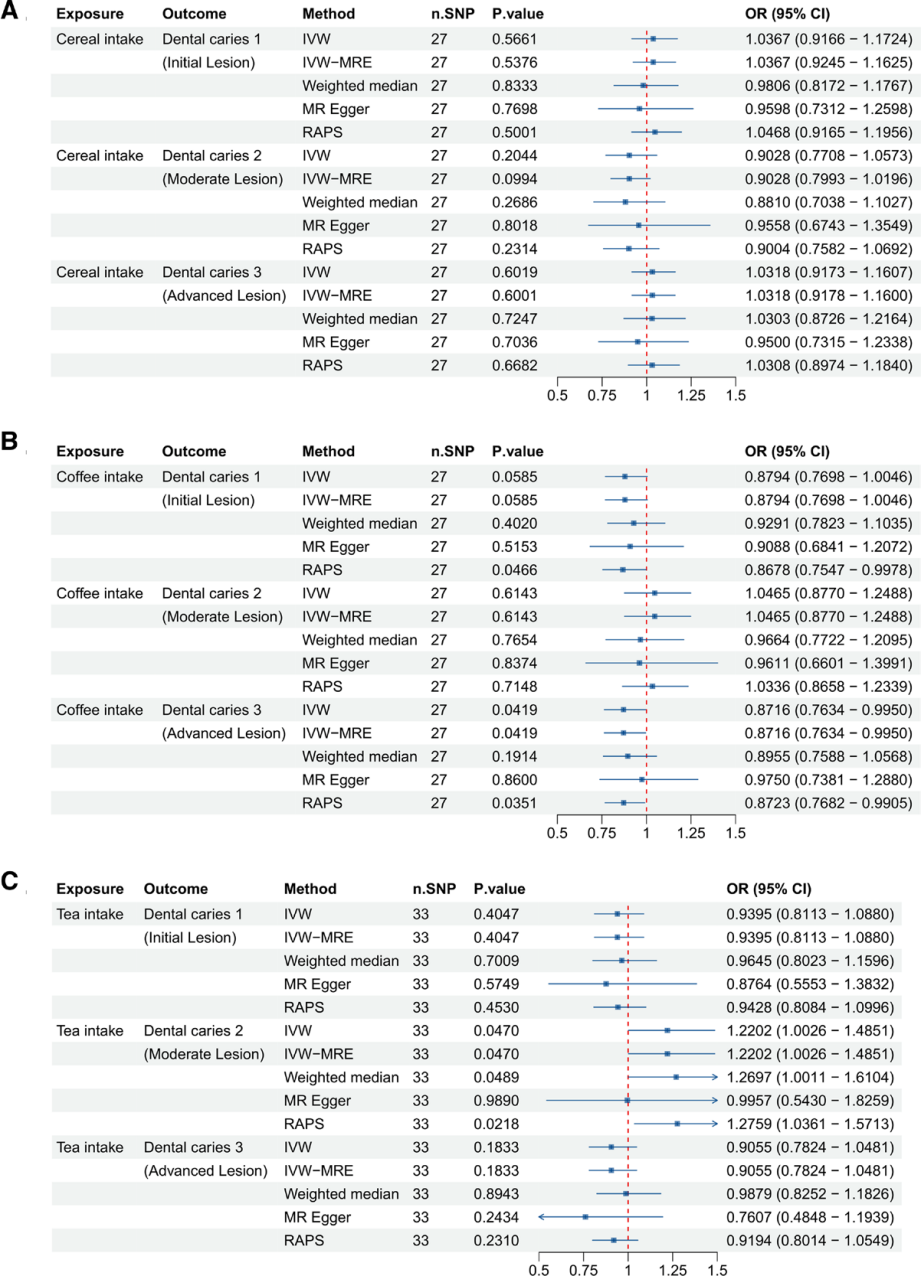
Forest plots illustrating the odds ratios (ORs) and 95% confidence intervals (CIs) for sugar intake in different contexts and dental caries risk using different Mendelian randomization (MR) methods. (A) Sugar added to cereal; (B) sugar added to coffee; and (C) sugar added to tea. Significant findings and the robustness of these associations are highlighted across methods such as IVW, IVW-MRE, weighted median, MR-Egger, and RAPS. CI = confidence interval, IVW = inverse-variance weighted, MR = Mendelian randomization, OR = odds ratio, RAPS = robust adjusted profile score, SNP = single nucleotide polymorphism.

We explored the causal relationship between sugar-related behaviors, such as adding sugar to food, and the development of dental caries (Fig. 4). For adding sugar to cereal (ukb-b-11697), no significant associations were found with any type of dental caries (1, 2, or 3), as indicated by *P*-values > .05.

In contrast, adding sugar to coffee (ukb-b-11697) showed a borderline significant negative association with dental caries 1, confirmed as significant by the RAPS method, and a significant negative association with dental caries 3, suggesting a protective effect. However, there was no significant association with dental caries 2.

On the other hand, adding sugar to tea (ukb-b-8442), did not show a significant association with dental caries 1 or 3, but it was significantly positively associated with dental caries 2, as supported by both the IVW and RAPS methods, indicating an increased risk. These findings highlight that the impact of adding sugar varies by the beverage type and specific dental caries outcome, with sugar in tea potentially increasing risk for dental caries 2, while sugar in coffee may reduce the risk for dental caries 3.

### 3.5. The practice of adding artificial sweeteners to food and dental caries

**Figure 5.**
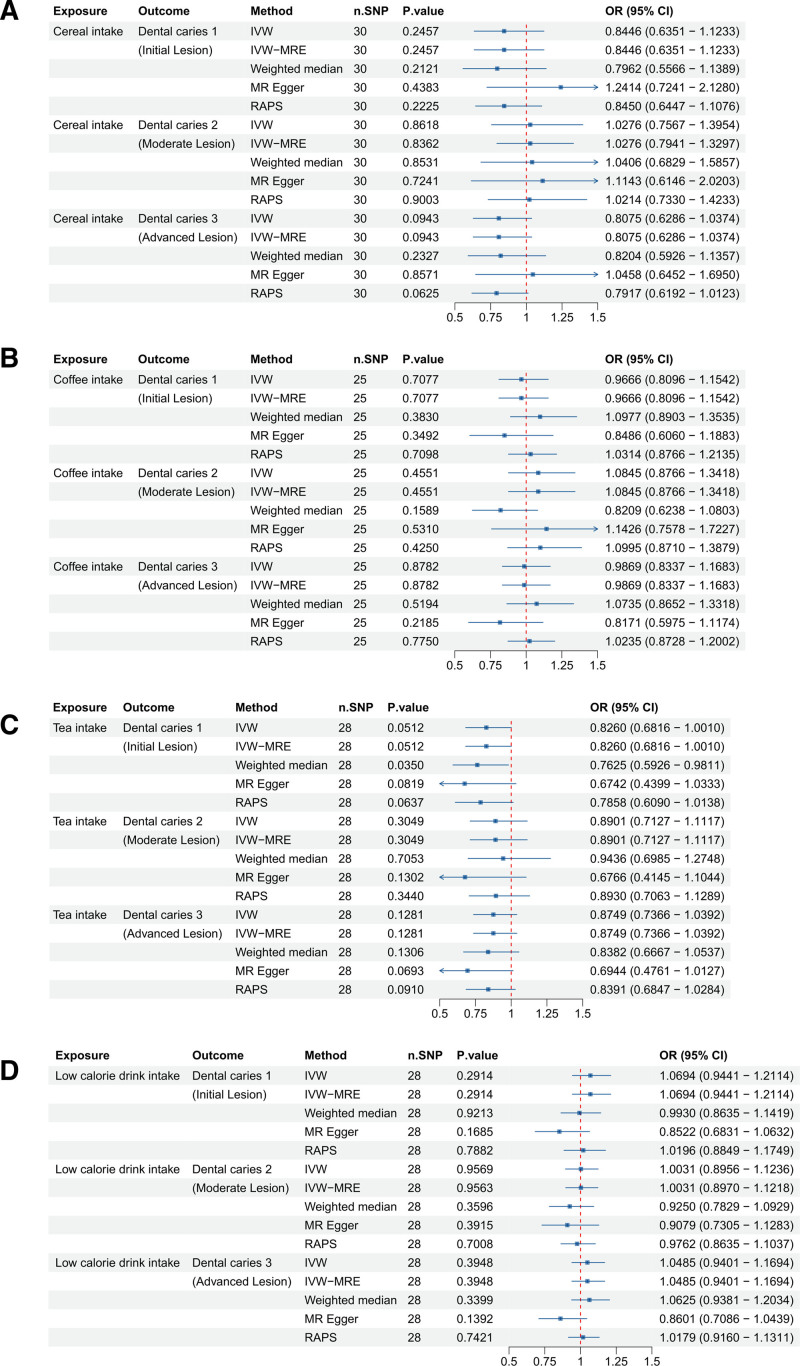
Forest plots illustrating the odds ratios (ORs) and 95% confidence intervals (CIs) for artificial sweetener intake in different contexts and low-calorie drink consumption related to dental caries risk using different Mendelian randomization (MR) methods. (A) Artificial sweetener added to cereal; (B) artificial sweetener added to coffee; (C) artificial sweetener added to tea; and (D) low-calorie drink intake. Significant findings and the robustness of these associations are highlighted across methods such as IVW, IVW-MRE, weighted median, MR-Egger, and RAPS. CI = confidence interval, IVW = inverse-variance weighted, MR = Mendelian randomization, OR = odds ratio, RAPS = robust adjusted profile score, SNP = single nucleotide polymorphism.

We investigated the causal relationship between sugar-related behaviors, such as adding artificial sweeteners to food, and the development of dental caries (Fig. 5). For artificial sweetener-added cereal (ukb-b-3143), no significant associations were found with dental caries 1, 2, or 3, as indicated by *P*-values > .05. Similarly, artificial sweetener-added coffee (ukb-b-1338), showed no significant associations with any type of dental caries.

In contrast, artificial sweetener-added tea (ukb-b-5867) bordered on a significant negative association with dental caries 1, supported by other methods like IVW-MRE and the weighted median, suggesting a strong protective effect, although no significant associations were found for dental caries 2 and 3. Lastly, low-calorie drink intake (ukb-b-5867) did not show significant associations with any dental caries outcomes, indicating that the addition of artificial sweeteners generally does not increase the risk of dental caries, with some potential protective effects observed for sweetened tea.

### 3.6. Fruits containing presumably high sucrose levels and dental caries

**Figure 6.**
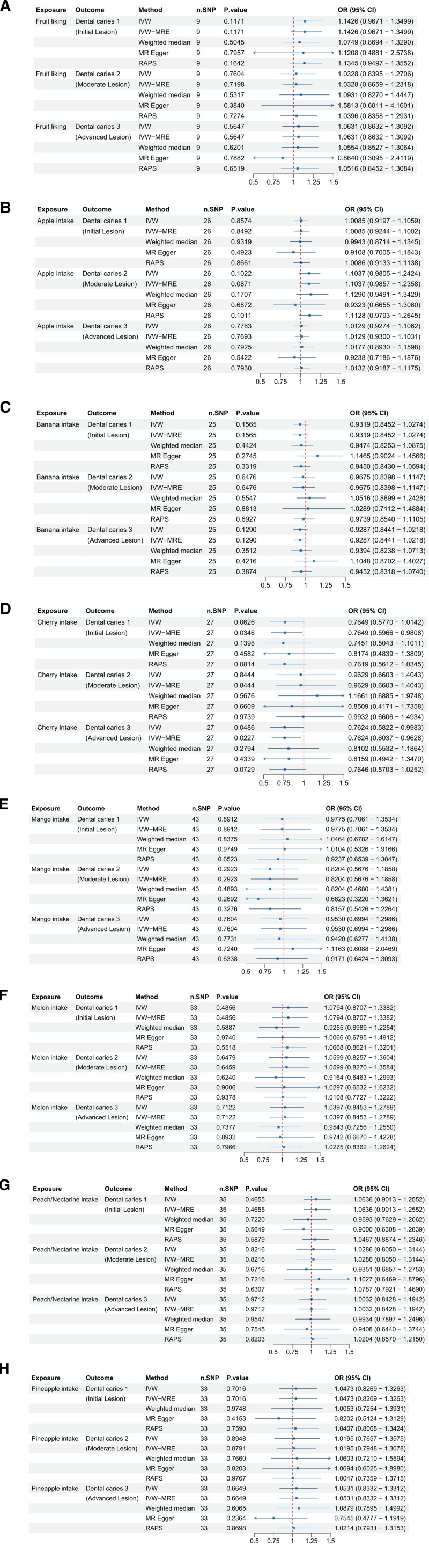
Forest plots illustrating the odds ratios (ORs) and 95% confidence intervals (CIs) for fruit preferences and specific fruit intakes related to dental caries risk using different Mendelian randomization (MR) methods. (A) Fruit liking; (B) apple intake; (C) banana intake; (D) cherry intake; (E) mango intake; (F) melon intake; (G) peach/nectarine intake; and (H) pineapple intake. Significant findings and the robustness of these associations are highlighted across methods such as IVW, IVW-MRE, weighted median, MR-Egger, and RAPS. CI = confidence interval, IVW = inverse-variance weighted, MR = Mendelian randomization, OR = odds ratio, RAPS = robust adjusted profile score, SNP = single nucleotide polymorphism.

We investigated the causal relationship between fruits containing presumably high sucrose levels and the development of dental caries (Fig. 6). Across various fruits, including apples (ukb-b-4070), bananas (ukb-b-5362), mangoes (ukb-b-19862), melons (ukb-b-6218), peaches/nectarines (ukb-b-6154), and pineapples (ukb-b-1164), no significant associations were found with dental caries 1, 2, or 3, as indicated by *P*-values > .05. However, cherry intake (ukb-b-2221) showed a borderline significant negative association with dental caries 1, confirmed as significant by the IVW-MRE method, and a significant negative association with dental caries 3, suggesting a protective effect. Fruit liking in general also showed no significant associations across all dental caries types.^[27]^ These findings indicate that while most fruits examined do not significantly impact dental caries risk, cherries may offer some protective benefits against certain types of dental caries.

### 3.7. Glucose level & diabetes and dental caries

**Figure 7.**
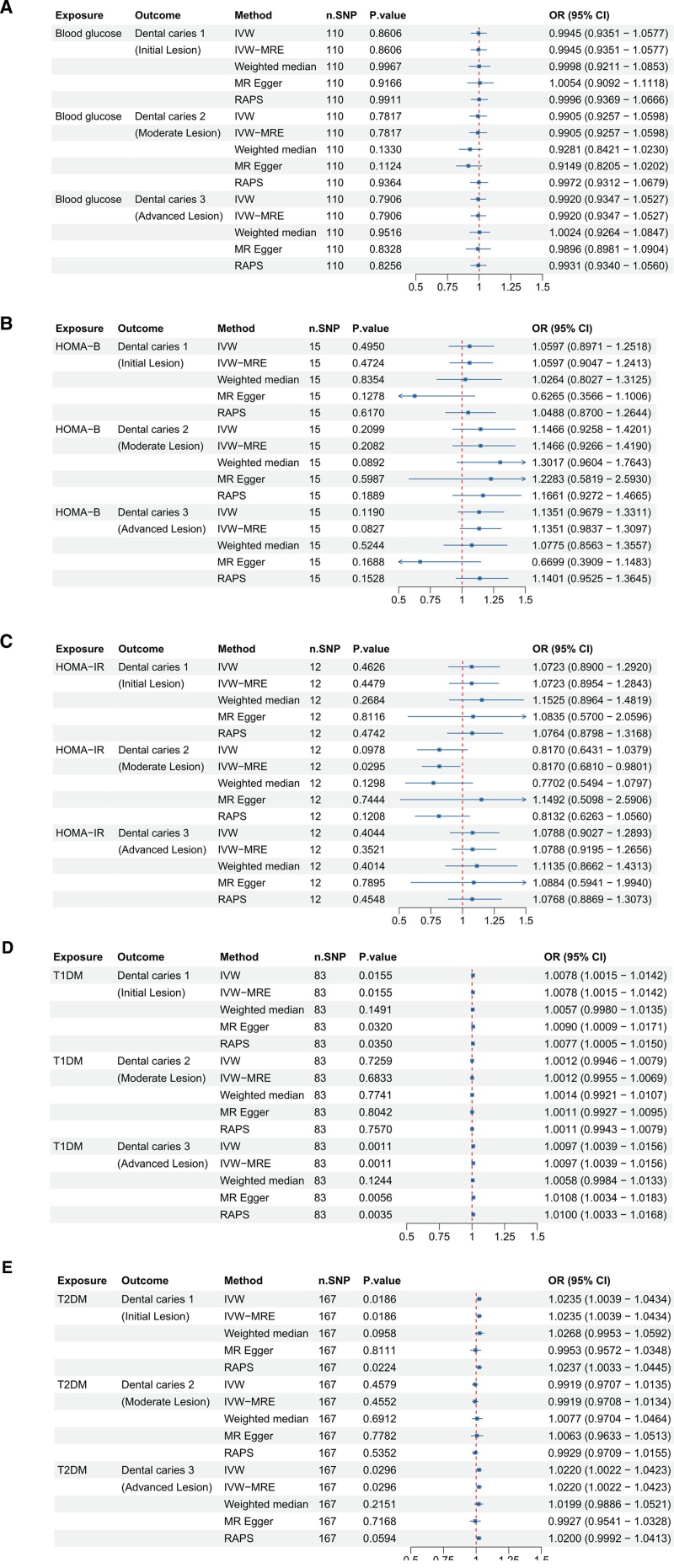
Forest plots illustrating the odds ratios (ORs) and 95% confidence intervals (CIs) for various metabolic and diabetes-related factors associated with dental caries risk using different Mendelian randomization (MR) methods. (A) Blood glucose level; (B) HOMA-B; (C) HOMA-IR; (D) type 1 diabetes mellitus (T1DM); and (E) type 2 diabetes mellitus (T2DM). Significant findings and the robustness of these associations are highlighted across methods such as IVW, IVW-MRE, weighted median, MR-Egger, and RAPS. CI = confidence interval, IVW = inverse-variance weighted, MR = Mendelian randomization, OR = odds ratio, RAPS = robust adjusted profile score, SNP = single nucleotide polymorphism, T1DM = type 1 diabetes mellitus, T2DM = type 2 diabetes mellitus.

Finally, we analyzed clinical and laboratory data related to sugar intake to investigate its relationship with dental caries (Fig. 7). Blood glucose levels (ebi-a-GCST90025986) showed no significant associations with any type of dental caries.

Similarly, HOMA-B levels (ieu-b-117) did not exhibit significant associations with dental caries 1, 2, or 3. For HOMA-IR levels (ieu-b-118), no significant associations were found with dental caries 1 and 3, although a significant negative association with dental caries 2 was suggested by the IVW-MRE method.

Type 1 diabetes mellitus (T1DM; ebi-a-GCST90014023) demonstrated significant positive associations with dental caries 1 and 3, as confirmed by multiple methods, indicating a strong relationship. Type 2 diabetes mellitus (T2DM; ebi-a-GCST90014023) was significantly positively associated with dental caries 1 and 3, suggesting a connection, though results for dental caries 2 were not significant. These findings suggest that while general blood glucose and insulin resistance markers may not directly correlate with dental caries, diabetes, particularly T1DM and T2DM, is strongly associated with an increased risk of certain types of dental caries. In secondary analyses, we explored associations between genetically proxied sugar-related exposures and indices of insulin sensitivity and resistance (HOMA-IR and HOMA-B). We observed no consistent evidence supporting a causal association, though a nominally significant negative association was detected between HOMA-IR and dental caries 2. This result should be interpreted cautiously, as it may reflect chance findings or phenotype-specific variation rather than a true biological effect.

### 3.8. Sugar and diabetes

Having confirmed a strong correlation between T1DM, T2DM, and dental caries, we investigated whether all previously studied sugar-related data were associated with T1D and T2D. However, none of the sugar-related exposures (such as chocolate foods, sugar-rich foods, the practice of adding sugar to food, the practice of adding artificial sweeteners to food, and fruits containing presumably high sucrose levels) showed a significant relationship with the outcomes of T1D and T2D. The results are presented in Figure S1, Supplemental Digital Content, https://links.lww.com/MD/Q893. These findings suggest that sugar-related dietary habits do not have an impact on diabetes.

## 4. Discussion

This study applied MR to investigate the genetic and dietary influences on dental caries. Contrary to previous assumptions, sugar preference did not significantly increase dental caries experience, suggesting that genetic predisposition and metabolic factors may play a more substantial role.^[2,28,29]^ Our findings suggest that genetically proxied sugar preference may not have a strong direct causal effect on dental caries risk, but this does not discount the established importance of environmental and behavioral factors, such as oral hygiene and fluoride exposure. Instead our findings support a more nuanced interpretation, where genetic predisposition, salivary characteristics, and metabolic conditions may exert stronger effects on caries development than sugar preference alone.

Although prior research has indicated shared genetic factors between diabetes and dental caries, our findings suggest that sugar preference may not be a primary genetic mediator in this relationship. The lack of genetic correlation may reflect indirect pathways or pleiotropic effects rather than a direct causal link. Furthermore, the null genetic correlation between sugar preference and diabetes does not necessarily contradict prior evidence of shared etiology; differences in methodology (polygenic overlap vs MR) and the possibility of indirect mechanisms likely explain these differences.

To validate our methodological approach, smoking status was employed as a positive control due to its well-established detrimental effects on oral health (Fig. 1). The literature consistently indicates that smokers have a 1.5 to 2 times higher risk of developing dental caries compared to nonsmokers.^[30]^ Furthermore, smoking has been shown to increase the activity of caries-related microorganisms by over 30%.^[31]^ A longitudinal study spanning 4 years reinforced this, revealing a 2.5 times higher incidence of dental caries among smokers.^[32]^ Smoking status was chosen as a positive control because its detrimental effects on oral health are well-established and biologically distinct from sugar consumption, primarily involving altered immune function, salivary composition, and oral microbiome dysbiosis. While lifestyle factors often co-occur, we confirmed that the genetic instruments for sugar preference were not associated with smoking status and found robust associations between smoking and dental caries in our MR analysis, supporting its validity as a positive control outcome. The robust associations between smoking and dental caries observed in our MR analysis support the instrument strength and reinforce the plausibility of our causal inference framework, though results must still be interpreted cautiously.

### 4.1. Genetic and environmental factors in dental caries

Among sugar-containing foods, chocolate and other sugar-added foods exposures did not show significant associations with caries outcomes, with the exception of cake, which was positively associated with enamel decay and pulp involvement.^[33,34]^ This finding suggests that while certain sugar-rich foods may contribute to dental caries, their overall effect is likely dependent on additional environmental and genetic factors. Similarly, the consumption of beverages with added sugar did not consistently correlate with an increased risk of dental caries.^[34]^ In contrast, sugar added to coffee showed a negative association with caries, possibly due to the antimicrobial effects of coffee components, which may counteract the cariogenic effects of sugar.^[35,36]^ Artificial sweeteners, which have been suggested as a safer alternative to sugar, generally did not show significant associations with dental caries, with the exception of tea sweeteners, which exhibited a weak negative correlation.^[37,38]^ Additionally, among various fruits, cherry intake was found to be inversely associated with dental caries, supporting previous findings on the protective effects of certain bioactive compounds in fruits.^[39]^ These protective associations with milk chocolate and cherry consumption should be interpreted with caution. While milk chocolate may contain dairy components that buffer acids and provide calcium, and cherries may contain polyphenols with anticariogenic properties, these findings could also reflect unmeasured lifestyle factors or residual confounding. Importantly, our results do not suggest that increased consumption of these foods should be encouraged, as public health guidelines continue to recommend limiting free sugar intake based on robust evidence of its detrimental effects on oral and systemic health.

### 4.2. Diabetes and dental caries

One of the most significant findings of this study was the strong association between both T1DM and T2DM and dental caries 1 and 3. The observed association between diabetes and dental caries may be mediated by diabetes-induced changes in salivary composition, immune dysfunction, or microbiome shifts, rather than reflecting a direct effect of glucose metabolism on tooth structures. Hyperglycemia-induced alterations in salivary flow and composition (elevated glucose levels, reduced buffering capacity), immune dysregulation, and shifts in the oral microbiome may increase cariogenic bacterial growth and impair enamel remineralization. These mechanisms may explain why T1DM and T2DM were both strongly associated with increased dental caries risk, despite the lack of a direct association with fasting blood glucose levels.

Our findings suggest that metabolic and genetic predispositions may influence dental caries risk in part through their impact on the oral microbiome. For example, diabetes-induced changes in salivary glucose and immune function, or sugar-rich dietary patterns influenced by genetic sweet preference, may promote a cariogenic microbiome. Further studies integrating metagenomic profiling are warranted.

Both type 1 and type 2 diabetes were significantly correlated with increased dental caries experience.^[40–43]^ Notably, the results indicate that individuals diagnosed with diabetes often modify their dietary habits, reducing sugar intake after diagnosis.^[27,44]^ This suggests that metabolic dysregulation may contribute more significantly to caries susceptibility in individuals with diabetes. The isolated negative association observed between HOMA-IR and dental caries 2 may reflect chance findings or phenotype-specific factors, such as differences in lesion progression rates or dietary behaviors among individuals with varying insulin resistance levels. This finding should be interpreted cautiously and warrants further investigation. These exploratory findings suggest that sugar preference, as proxied by genetic instruments, may not directly influence insulin sensitivity or β-cell function. However, the complex interplay between dietary sugar intake, insulin resistance, and downstream metabolic outcomes warrants further mechanistic investigation in future studies.

Our MR analysis did not detect a statistically significant causal relationship between genetically proxied sugar-related dietary habits and diabetes risk. This result should be interpreted with caution and in light of differences in study design compared to prior observational studies. Traditional epidemiologic studies often rely on self-reported dietary data and are inherently susceptible to residual confounding and reverse causation, which can overestimate associations. By contrast, MR estimates the effect of a genetically influenced tendency toward sugar preference, which may not fully capture the behavioral and environmental determinants of total sugar intake. Therefore, the apparent discrepancy between our findings and prior evidence may reflect differences in methodology and the fact that genetically proxied sugar preference does not equate to actual sugar consumption patterns. Future studies combining genetic, behavioral, and longitudinal dietary data will be essential to further clarify this relationship.

### 4.3. Reconciling MR findings with observational studies

The discrepancy between our findings and observational studies likely arises due to methodological differences: observational research captures the combined effects of dietary behavior and multiple environmental confounders, whereas our MR study isolates the genetically driven preference for sweetness. This distinction highlights the necessity for interpreting MR results within a broader context, emphasizing that genetic predisposition alone does not fully represent dietary behavior’s impact on caries development.

### 4.4. Limitations and future research directions

#### 4.4.1. Use of GWAS summary statistics

This study utilized GWAS summary statistics, which, while valuable, have inherent limitations in controlling for confounders. Although the use of MR helps mitigate issues related to reverse causation and confounding, residual biases may still be present.^[45–47]^ Although the MR design mitigates confounding by behavioral and socioeconomic factors, residual confounding from unmeasured variables such as diet quality, physical activity, or access to dental care cannot be fully excluded. Our null findings for sugar preference and dental caries should also be interpreted in light of potential measurement limitations. Although we used robust MR methodology, the study design and genetic instruments may not have been sufficiently sensitive to detect modest associations. Genetically predicted sugar preference may not fully capture the behavioral and environmental determinants of actual sugar consumption, which could attenuate observed associations.

#### 4.4.2. Population generalizability

The data used in this study were primarily derived from European ancestry cohorts, which may limit the generalizability of the findings to other populations. Future research should aim to include more diverse populations to ensure broader applicability.^[46–48]^ Cultural differences in sugar consumption patterns, oral hygiene practices, and access to dental care across populations may also influence the observed associations. Therefore, the extent to which these findings apply to non-European populations remains uncertain, and validation in more diverse cohorts is required.

#### 4.4.3. Potential pleiotropy

Another limitation involves the potential influence of pleiotropy. While statistical approaches were applied to detect and correct for horizontal pleiotropy, it cannot be entirely ruled out as a confounding factor in the results.^[49,50]^ These limitations related to potential horizontal pleiotropy and instrument availability may bias causal estimates and therefore should be interpreted with caution. To further ensure the robustness of our findings, we used a combination of MR methods, including IVW, IVW with multiplicative random effects (IVW-MRE), weighted median, and RAPS. These complementary approaches are designed to be robust against potential violations of MR assumptions. In addition, we applied false discovery rate correction across all exposure-outcome associations to minimize the risk of false positives due to multiple testing.

#### 4.4.4. Weak instrument bias

All IVs used in this study had *F*-statistics > 10, with a minimum value of 19.62, confirming that weak instrument bias is unlikely. However, despite these statistical safeguards, the reliance on genetic instruments still presents inherent limitations in capturing the full complexity of gene–environment interactions.

#### 4.4.5. Lack of individual-level data

The study design also lacked longitudinal data, which restricts the ability to assess the long-term impact of sugar preference on dental caries experience. Future studies should incorporate prospective cohort designs to provide more robust evidence on the causal pathways linking dietary habits, metabolic conditions, and dental caries.^[51]^ While our MR analysis provides insight into the potential causal role of sugar preference, we recognize that dental caries is a multifactorial condition, and our study does not capture the impact of critical confounders such as fluoride exposure, oral hygiene, and access to dental care.

##### 4.4.5.1. Exposure classification

In this study, exposures were grouped based on dietary content and UK Biobank field similarity. “Chocolate foods” included chocolate bar (ukb-b-117), chocolate-covered biscuits (ukb-b-5068), milk chocolate (ukb-b-4569), chocolate sweets (ukb-b-9835), and others. “Sugar-rich foods” included items such as cake (ukb-b-3433), doughnut (ukb-b-6214), and fizzy drinks (ukb-b-2832). “Added sugar” behaviors included sugar added to coffee (ukb-b-11697), tea (ukb-b-8442), and cereal (ukb-b-11697), and “Artificial sweeteners” referred to sweetener use in tea (ukb-b-5867), coffee (ukb-b-1338), or cereal (ukb-b-3143). These classifications were defined based on prior nutritional profiling and categorized for interpretability within MR analysis.

##### 4.4.5.2. SNP summary statistics

For each exposure, we selected genome-wide significant SNPs (*P* < 5 × 10⁻⁸) and applied LD clumping (*r*² < 0.001) to ensure independence. For example, chocolate bar intake (ukb-b-117) initially had 34 SNPs, which were pruned to 21 after LD filtering. Of these, 2 SNPs were excluded due to prior associations with dental caries or periodontitis, leaving 19 final SNPs for MR analysis. The SNP count across exposures ranged from 5 to 29 after filtering.

While our MR analysis provides insight into the potential causal role of sugar preference, we recognize that dental caries is a multifactorial condition, and our study does not capture the impact of critical confounders such as fluoride exposure, oral hygiene, and access to dental care.

Our study highlights several critical knowledge gaps regarding the complex interactions among genetic predispositions, lifestyle behaviors, and oral health outcomes. Dental caries arises from complex interactions among genetic susceptibility, environmental exposures (e.g., fluoride availability, oral hygiene behaviors), and metabolic conditions. These factors may influence one another, for example through shared effects on salivary composition or oral microbiome diversity. Future studies should specifically investigate how genetic variants related to sweet taste preference and metabolic conditions shape oral microbiome composition and function, as this could provide deeper insight into caries pathogenesis and potential preventive strategies. Longitudinal cohort designs integrating genomic, metagenomic, and metabolomic analyses will be particularly valuable for clarifying these mechanisms. Additionally, interventional studies examining how dietary modifications or enhanced oral hygiene practices influence microbiome dynamics and dental caries risk in genetically susceptible or metabolically at risk populations are warranted. Larger GWAS datasets and expanded analyses in multiethnic cohorts will be crucial to improve instrument strength and mitigate biases related to sample size and population structure inherent to the current study design.

These findings may offer preliminary insights into how individual genetic and metabolic profiles could be considered in future personalized dental care models, though further validation is necessary before clinical implementation. Our findings highlight the need for future research to explore the interaction between genetic predispositions and lifestyle factors in oral health. Key areas for investigation include the influence of genetic variants on the oral microbiome and the mediating role of diabetes-related changes in salivary conditions. We recommend using integrated multi-omics analyses, longitudinal cohort studies, and interventional trials to unravel these complex interactions and guide personalized prevention strategies.

## 5. Conclusion

This study provides novel insights that challenge the traditional paradigm linking sugar preference directly to dental caries. While some sugar-rich dietary traits (e.g., cake) were associated with increased risk, genetic and metabolic factors – including diabetes – appear to have a stronger and more consistent relationship with caries outcomes. Furthermore, the lack of significant associations between sugar intake traits and diabetes suggests that the caries–diabetes link may not be directly mediated by sugar exposure alone. These results should be interpreted as indicating potential trends rather than definitive causal conclusions, especially given methodological and biological complexities. Future research should apply multivariable MR and longitudinal designs in diverse populations to further clarify these complex relationships.

## Acknowledgments

Acknowledgment is given to the participants and investigators of the FinnGen study. Gratitude is also extended to the IEU Open-GWAS project for generously sharing the summary-level data. The MEGASTROKE project received funding from sources specified at https://www.megastroke.org/.

## Author contributions

**Conceptualization:** Sun Woo Lim, Junhua Wu, Zhihuan Tian, Seongjin Lim, Dong Woon Kim.

**Data curation:** Sun Woo Lim, Junhua Wu, Yeon Woo Kim, Seongjin Lim.

**Formal analysis:** Seung Gyu Choi, Hyewon Park.

**Supervision:** Yeon Woo Kim.

**Writing** – **original draft:** Sun Woo Lim.

**Writing** – **review & editing:** Joon Won Kang, Jin-Young Choi, Dong Woon Kim.

## Supplementary Material


