# Supplementary material for: Sugar consumption and its role in dental caries: Insights from a 2-sample Mendelian randomization study
Source: Medicine (Baltimore). 2025 Dec 19;104(51):e46479. doi: 10.1097/MD.0000000000046479 (PMC12727404; doi:10.1097/MD.0000000000046479)

Sugar Consumption and Its Role in Dental Caries: Insights from a Two-Sample Mendelian Randomization Study

Authors

Sun Woo Lim[a,*], Junhua Wu[b,*], Zhihuan Tian[a], Yeon Woo Kim[a], Seongjin Lim[a], Seung Gyu Choi[a], Hyewon Park[c], Joon Won Kang[d], Jin-Young Choi[e], Dong Woon Kim[a,#]

Affiliations

[a]Department of Oral Anatomy & Neurobiology, Kyung Hee University College of Dentistry, Seoul 02447, Republic of Korea

[b]Department of Neurology, Southwest Hospital, Third Military Medical University (Army Medical University), Chongqing 400038, China.

[c]Department of Medical Science, Chungnam National University College of Medicine, Daejeon 35015, Republic of Korea

[d]Department of Pediatrics, Chungnam National University Hospital, Daejeon 35015, Republic of Korea

[e]Department of Orthodontics, Kyung Hee University College of Dentistry, Kyung Hee University Medical Center, Seoul 02447, Republic of Korea

* authors contributed equally

Table 1. A complete description of each GWAS dataset

**Exposure / Outcome**	**GWAS Source (Database)**	**Cohort / Study name**	**Sample size (n)**	**Population ancestry**	**GWAS ID / Reference**

**Chocolate intake (bar, biscuits, sweets, etc.)**	MRC-IEU GWAS Database	UK Biobank	64,949	White British (European)	ukb-b-117, ukb-b-9886, ukb-b-5068, ukb-b-16139, ukb-b-16449, ukb-b-4569, ukb-b-1160, ukb-b-9835
**Sugar-added foods (cake, doughnut, fizzy drinks, etc.)**	MRC-IEU GWAS Database	UK Biobank	64,949	White British (European)	ukb-b-3433, ukb-b-6214, ukb-b-2832, ukb-b-17775, ukb-b-17189, ukb-b-6500, ukb-b-10366, ukb-b-10217
**Sugar added to foods (cereal, coffee, tea)**	MRC-IEU GWAS Database	UK Biobank	64,949	White British (European)	ukb-b-11697, ukb-b-243, ukb-b-8442
**Artificial sweeteners (cereal, coffee, tea, low-calorie drinks)**	MRC-IEU GWAS Database	UK Biobank	64,949	White British (European)	ukb-b-3143, ukb-b-1338, ukb-b-5867, ukb-b-19703
**Fruit liking**	GWAS Catalog	Multiple cohorts	Not specified (~40,000)	Mixed European	GCST90094766
**Fruit intakes (apple, banana, mango, etc.)**	MRC-IEU GWAS Database	UK Biobank	64,949	White British (European)	ukb-b-4070, ukb-b-5362, ukb-b-2221, ukb-b-19862, ukb-b-6218, ukb-b-6154, ukb-b-1164
**Blood glucose**	MRC-IEU	UK Biobank	400,458	White British	ebi-a-

**level**	GWAS Database			(European)	GCST90025986
**HOMA-B / HOMA-IR**	MAGIC Consortium	Multiple cohorts	36,466	European	ieu-b-117, ieu-b-118
**Type 1 Diabetes Mellitus (T1DM)**	FinnGen + UCSD + Ulm Univ.	FinnGen R11 & meta-analysis	~520,580	Finnish / Mixed European	summary_stats_finngen_R11_T1D
**Type 2 Diabetes Mellitus (T2DM)**	BioBank Japan + UK Biobank + FinnGen	Multi-cohort meta-analysis	~490,089	Mixed European & Asian	summary_stats_finngen_R11_T2D
**Dental caries (Outcome 1–3)**	FinnGen R11	FinnGen	Up to 500,000	Finnish	finngen_R11_K11_CARIES_1–3_OPER_ONLYAVO
**Smoking status (Positive control)**	MRC-IEU GWAS Database	UK Biobank	468,170	White British (European)	ebi-a-GCST90029014

Supplementary Figure 1: Forest plots illustrating the odds ratios (ORs) and 95% confidence intervals (CIs) for various metabolic and diabetes-related factors associated with dental caries risk using different Mendelian Randomization (MR) methods. A. Blood glucose level; B. HOMA-B; C. HOMA-IR; D. Type 1 Diabetes Mellitus (T1DM); E. Type 2 Diabetes Mellitus (T2DM). Significant findings and the robustness of these associations are highlighted across methods such as IVW, IVW-MRE, Weighted Median, MR Egger, and RAPS.

A.

Exposure	Outcome	Method	n.SNP	P.value	OR (95% CI)
Chocolate bar intake	T1DM	IVW	21	0.9777	1.0079 (0.5808 − 1.7490)
		IVW−MRE	21	0.9777	1.0079 (0.5808 − 1.7490)
		Weighted median	21	0.3940	0.7510 (0.3888 − 1.4507)
		MR Egger	21	0.5521	1.3632 (0.4999 − 3.7171)
		RAPS	21	0.8439	0.9426 (0.5234 − 1.6975)
Chocolate bar intake	T2DM	IVW	21	0.4094	0.9521 (0.8473 − 1.0698)
		IVW−MRE	21	0.3088	0.9521 (0.8663 − 1.0465)
		Weighted median	21	0.2492	0.9073 (0.7689 − 1.0706)
		MR Egger	21	0.9496	0.9932 (0.8062 − 1.2236)
		RAPS	21	0.3790	0.9448 (0.8326 − 1.0722)
Chocolate biscuits intake	T1DM	IVW	13	0.8128	0.9495 (0.6184 − 1.4579)
		IVW−MRE	13	0.8128	0.9495 (0.6184 − 1.4579)
		Weighted median	13	0.9610	1.0129 (0.6065 − 1.6915)
		MR Egger	13	0.5159	1.4079 (0.5185 − 3.8232)
		RAPS	13	0.9209	1.0235 (0.6464 − 1.6207)
Chocolate biscuits intake	T2DM	IVW	13	0.2222	0.9439 (0.8603 − 1.0356)
		IVW−MRE	13	0.1876	0.9439 (0.8662 − 1.0286)
		Weighted median	13	0.3195	0.9326 (0.8128 − 1.0700)
		MR Egger	13	0.3458	0.8984 (0.7259 − 1.1119)
		RAPS	13	0.2651	0.9376 (0.8371 − 1.0501)
Chocolate sweets intake	T1DM	IVW	26	0.9122	1.0270 (0.6394 − 1.6496)
		IVW−MRE	26	0.9122	1.0270 (0.6394 − 1.6496)
		Weighted median	26	0.9020	0.9592 (0.4938 − 1.8629)
		MR Egger	26	0.1329	0.4881 (0.1977 − 1.2050)
		RAPS	26	0.9576	0.9857 (0.5799 − 1.6755)
Chocolate sweets intake	T2DM	IVW	26	0.5435	0.9456 (0.7895 − 1.1326)
		IVW−MRE	26	0.5435	0.9456 (0.7895 − 1.1326)
		Weighted median	26	0.1551	0.8676 (0.7133 − 1.0552)
		MR Egger	26	0.8120	1.0459 (0.7257 − 1.5072)
		RAPS	26	0.8654	0.9849 (0.8260 − 1.1744)
Chocolate−covered biscuits intake	T1DM	IVW	25	0.1767	1.2970 (0.8894 − 1.8913)
		IVW−MRE	25	0.1762	1.2970 (0.8898 − 1.8905)
		Weighted median	25	0.6340	1.1453 (0.6551 − 2.0024)
		MR Egger	25	0.1705	1.9696 (0.7702 − 5.0370)
		RAPS	25	0.1528	1.3873 (0.8857 − 2.1729)
Chocolate−covered biscuits intake	T2DM	IVW	25	0.6264	1.0311 (0.9114 − 1.1665)
		IVW−MRE	25	0.6264	1.0311 (0.9114 − 1.1665)
		Weighted median	25	0.1437	1.1185 (0.9626 − 1.2997)
		MR Egger	25	0.1079	0.7816 (0.5855 − 1.0432)
		RAPS	25	0.3722	1.0605 (0.9321 − 1.2067)
Chocolate−covered raisin intake	T1DM	IVW	72	0.7079	1.1327 (0.5904 − 2.1731)
		IVW−MRE	72	0.7071	1.1327 (0.5915 − 2.1690)
		Weighted median	72	0.7619	0.8452 (0.2848 − 2.5086)
		MR Egger	72	0.8863	0.9084 (0.2443 − 3.3768)
		RAPS	72	0.6286	1.2120 (0.5561 − 2.6418)
Chocolate−covered raisin intake	T2DM	IVW	72	0.9262	1.0125 (0.7789 − 1.3161)
		IVW−MRE	72	0.9262	1.0125 (0.7789 − 1.3161)
		Weighted median	72	0.7822	0.9561 (0.6952 − 1.3148)
		MR Egger	72	0.2979	1.3250 (0.7831 − 2.2418)
		RAPS	72	0.7021	1.0611 (0.7829 − 1.4383)
Dark chocolate intake	T1DM	IVW	32	0.3512	1.3962 (0.6922 − 2.8163)
		IVW−MRE	32	0.3512	1.3962 (0.6922 − 2.8163)
		Weighted median	32	0.4956	1.3463 (0.5724 − 3.1665)
		MR Egger	32	0.8537	0.9010 (0.3003 − 2.7029)
		RAPS	32	0.5646	1.1929 (0.6545 − 2.1742)
Dark chocolate intake	T2DM	IVW	32	0.0054	1.3301 (1.0877 − 1.6264)
		IVW−MRE	32	0.0054	1.3301 (1.0877 − 1.6264)
		Weighted median	32	0.3307	1.1441 (0.8723 − 1.5005)
		MR Egger	32	0.5922	1.0882 (0.8013 − 1.4780)
		RAPS	32	0.0314	1.3250 (1.0254 − 1.7122)
Milk chocolate intake	T1DM	IVW	25	0.2352	1.4083 (0.8002 − 2.4785)
		IVW−MRE	25	0.1968	1.4083 (0.8373 − 2.3686)
		Weighted median	25	0.1298	1.8585 (0.8335 − 4.1439)
		MR Egger	25	0.5965	1.4269 (0.3896 − 5.2255)
		RAPS	25	0.2003	1.4922 (0.8088 − 2.7530)
Milk chocolate intake	T2DM	IVW	25	0.5302	0.9312 (0.7455 − 1.1633)
		IVW−MRE	25	0.5302	0.9312 (0.7455 − 1.1633)
		Weighted median	25	0.1234	0.8236 (0.6435 − 1.0542)
		MR Egger	25	0.1063	0.6531 (0.3975 − 1.0733)
		RAPS	25	0.1146	0.8410 (0.6782 − 1.0428)
Hot chocolate intake	T1DM	IVW	30	0.6956	1.1647 (0.5425 − 2.5007)
		IVW−MRE	30	0.6956	1.1647 (0.5425 − 2.5007)
		Weighted median	30	0.9083	0.9362 (0.3047 − 2.8758)
		MR Egger	30	0.9733	0.9728 (0.1958 − 4.8322)
		RAPS	30	0.6953	1.1767 (0.5213 − 2.6559)
Hot chocolate intake	T2DM	IVW	30	0.3172	0.8505 (0.6192 − 1.1681)
		IVW−MRE	30	0.3172	0.8505 (0.6192 − 1.1681)
		Weighted median	30	0.8911	1.0249 (0.7209 − 1.4570)
		MR Egger	30	0.4113	0.7539 (0.3882 − 1.4642)
		RAPS	30	0.2707	0.7864 (0.5128 − 1.2060)

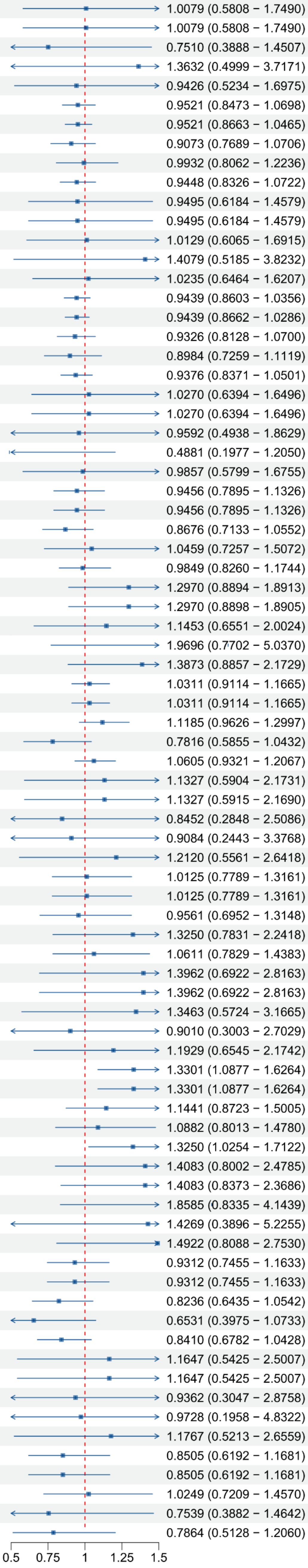

B.

Exposure	Outcome	Method	n.SNP	P.value	OR (95% CI)
Cake intake	T1DM	IVW	20	0.8264	0.9492 (0.5958 − 1.5123)
		IVW−MRE	20	0.8107	0.9492 (0.6196 − 1.4541)
		Weighted median	20	0.4322	1.3319 (0.6514 − 2.7232)
		MR Egger	20	0.6644	0.8343 (0.3731 − 1.8660)
		RAPS	20	0.9457	0.9826 (0.5934 − 1.6271)
Cake intake	T2DM	IVW	20	0.1080	0.8945 (0.7808 − 1.0248)
		IVW−MRE	20	0.1080	0.8945 (0.7808 − 1.0248)
		Weighted median	20	0.0381	0.8189 (0.6781 − 0.9891)
		MR Egger	20	0.0888	0.8063 (0.6378 − 1.0194)
		RAPS	20	0.0790	0.8761 (0.7559 − 1.0154)
Doughnut intake	T1DM	IVW	51	0.5091	0.7393 (0.3016 − 1.8124)
		IVW−MRE	51	0.4693	0.7393 (0.3262 − 1.6755)
		Weighted median	51	0.8351	1.1496 (0.3094 − 4.2705)
		MR Egger	51	0.5866	0.6427 (0.1320 − 3.1287)
		RAPS	51	0.3483	0.6293 (0.2391 − 1.6566)
Doughnut intake	T2DM	IVW	51	0.8488	0.9642 (0.6628 − 1.4026)
		IVW−MRE	51	0.8488	0.9642 (0.6628 − 1.4026)
		Weighted median	51	0.8759	1.0342 (0.6782 − 1.5770)
		MR Egger	51	0.4453	0.7707 (0.3970 − 1.4961)
		RAPS	51	0.8258	0.9545 (0.6305 − 1.4450)
Fizzy drink intake	T1DM	IVW	23	0.6553	1.1407 (0.6400 − 2.0332)
		IVW−MRE	23	0.6553	1.1407 (0.6400 − 2.0332)
		Weighted median	23	0.6337	1.1841 (0.5910 − 2.3722)
		MR Egger	23	0.4064	1.6830 (0.5047 − 5.6118)
		RAPS	23	0.5554	1.1792 (0.6818 − 2.0396)
Fizzy drink intake	T2DM	IVW	23	0.9542	0.9943 (0.8173 − 1.2096)
		IVW−MRE	23	0.9542	0.9943 (0.8173 − 1.2096)
		Weighted median	23	0.2030	0.8763 (0.7150 − 1.0739)
		MR Egger	23	0.5725	0.8872 (0.5893 − 1.3358)
		RAPS	23	0.6257	0.9572 (0.8029 − 1.1411)
Fruit cake intake	T1DM	IVW	25	0.7500	0.8707 (0.3716 − 2.0405)
		IVW−MRE	25	0.7500	0.8707 (0.3716 − 2.0405)
		Weighted median	25	0.9870	1.0104 (0.2912 − 3.5060)
		MR Egger	25	0.2805	3.6253 (0.3694 − 35.5835)
		RAPS	25	0.7904	0.8836 (0.3549 − 2.1999)
Fruit cake intake	T2DM	IVW	25	0.5923	1.1307 (0.7213 − 1.7724)
		IVW−MRE	25	0.5923	1.1307 (0.7213 − 1.7724)
		Weighted median	25	0.4790	0.8687 (0.5884 − 1.2826)
		MR Egger	25	0.5228	1.5022 (0.4395 − 5.1344)
		RAPS	25	0.5006	1.1847 (0.7234 − 1.9401)
Ice−cream intake	T1DM	IVW	29	0.9941	0.9976 (0.5238 − 1.8999)
		IVW−MRE	29	0.9941	0.9976 (0.5238 − 1.8999)
		Weighted median	29	0.9985	0.9992 (0.4534 − 2.2023)
		MR Egger	29	0.4024	1.9605 (0.4156 − 9.2470)
		RAPS	29	0.7595	1.1069 (0.5778 − 2.1204)
Ice−cream intake	T2DM	IVW	29	0.4149	0.9169 (0.7443 − 1.1295)
		IVW−MRE	29	0.4149	0.9169 (0.7443 − 1.1295)
		Weighted median	29	0.9817	0.9971 (0.7816 − 1.2721)
		MR Egger	29	0.3614	0.7870 (0.4748 − 1.3048)
		RAPS	29	0.5596	0.9259 (0.7149 − 1.1992)
Pancake intake	T1DM	IVW	72	0.5722	0.7892 (0.3472 − 1.7942)
		IVW−MRE	72	0.5722	0.7892 (0.3472 − 1.7942)
		Weighted median	72	0.9364	0.9518 (0.2827 − 3.2046)
		MR Egger	72	0.5313	0.6290 (0.1484 − 2.6664)
		RAPS	72	0.7702	0.8746 (0.3558 − 2.1494)
Pancake intake	T2DM	IVW	72	0.3238	1.1664 (0.8592 − 1.5834)
		IVW−MRE	72	0.3238	1.1664 (0.8592 − 1.5834)
		Weighted median	72	0.8975	1.0266 (0.6889 − 1.5298)
		MR Egger	72	0.2311	1.3913 (0.8142 − 2.3771)
		RAPS	72	0.1709	1.3152 (0.8885 − 1.9466)
Sweet snack consumer	T1DM	IVW	20	0.5914	1.2779 (0.5220 − 3.1287)
		IVW−MRE	20	0.5914	1.2779 (0.5220 − 3.1287)
		Weighted median	20	0.5533	0.7089 (0.2273 − 2.2107)
		MR Egger	20	0.5546	1.8430 (0.2519 − 13.4834)
		RAPS	20	0.5461	1.3311 (0.5260 − 3.3685)
Sweet snack consumer	T2DM	IVW	20	0.4113	0.8794 (0.6472 − 1.1949)
		IVW−MRE	20	0.4113	0.8794 (0.6472 − 1.1949)
		Weighted median	20	0.2250	0.7926 (0.5444 − 1.1538)
		MR Egger	20	0.7807	0.9060 (0.4569 − 1.7967)
		RAPS	20	0.2345	0.8300 (0.6105 − 1.1284)
Sweets intake	T1DM	IVW	35	0.4983	1.1257 (0.7990 − 1.5861)
		IVW−MRE	35	0.4600	1.1257 (0.8222 − 1.5413)
		Weighted median	35	0.7021	1.1022 (0.6694 − 1.8147)
		MR Egger	35	0.4510	1.2832 (0.6762 − 2.4350)
		RAPS	35	0.3111	1.2114 (0.8358 − 1.7559)
Sweets intake	T2DM	IVW	35	0.9081	0.9929 (0.8802 − 1.1201)
		IVW−MRE	35	0.9081	0.9929 (0.8802 − 1.1201)
		Weighted median	35	0.9262	1.0069 (0.8711 − 1.1638)
		MR Egger	35	0.4514	0.9157 (0.7301 − 1.1485)
		RAPS	35	0.9597	0.9973 (0.8987 − 1.1068)

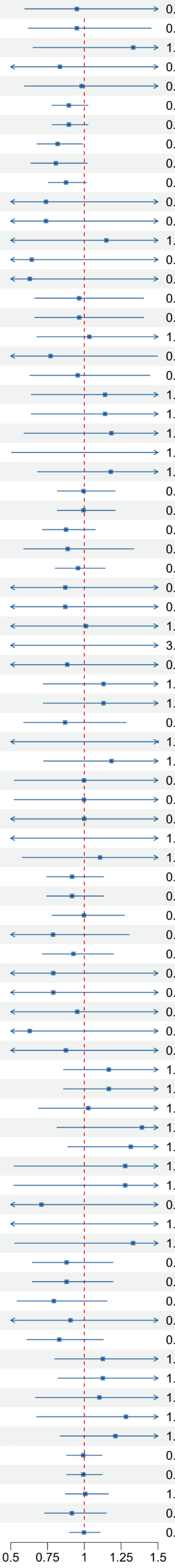

C.

Exposure	Outcome	Method	n.SNP	P.value	OR (95% CI)
Practice of adding sugar to cereal	T1DM	IVW	27	0.8933	0.9704 (0.6261 − 1.5042)
		IVW−MRE	27	0.8933	0.9704 (0.6261 − 1.5042)
		Weighted median	27	0.1616	0.6530 (0.3596 − 1.1860)
		MR Egger	27	0.6712	0.8066 (0.3025 − 2.1507)
		RAPS	27	0.8361	0.9499 (0.5837 − 1.5457)
Practice of adding sugar to cereal	T2DM	IVW	27	0.1273	1.0985 (0.9735 − 1.2396)
		IVW−MRE	27	0.1273	1.0985 (0.9735 − 1.2396)
		Weighted median	27	0.5843	1.0503 (0.8810 − 1.2520)
		MR Egger	27	0.6641	0.9424 (0.7232 − 1.2280)
		RAPS	27	0.1613	1.0974 (0.9636 − 1.2498)
Practice of adding sugar to coffee	T1DM	IVW	27	0.2635	0.7771 (0.4995 − 1.2091)
		IVW−MRE	27	0.2635	0.7771 (0.4995 − 1.2091)
		Weighted median	27	0.7043	1.1208 (0.6220 − 2.0195)
		MR Egger	27	0.7520	0.8579 (0.3352 − 2.1961)
		RAPS	27	0.3373	0.7885 (0.4852 − 1.2812)
Practice of adding sugar to coffee	T2DM	IVW	27	0.1684	0.7518 (0.5010 − 1.1282)
		IVW−MRE	27	0.1684	0.7518 (0.5010 − 1.1282)
		Weighted median	27	0.5115	0.9454 (0.7996 − 1.1179)
		MR Egger	27	0.6097	1.2475 (0.5394 − 2.8855)
		RAPS	27	0.2342	0.9204 (0.8029 − 1.0552)
Practice of adding sugar to tea	T1DM	IVW	33	0.6166	1.1139 (0.7303 − 1.6990)
		IVW−MRE	33	0.5975	1.1139 (0.7464 − 1.6623)
		Weighted median	33	0.5820	0.8487 (0.4731 − 1.5223)
		MR Egger	33	0.0855	3.2306 (0.8858 − 11.7825)
		RAPS	33	0.8224	1.0536 (0.6681 − 1.6615)
Practice of adding sugar to coffee	T2DM	IVW	33	0.0223	0.6263 (0.4192 − 0.9357)
		IVW−MRE	33	0.0223	0.6263 (0.4192 − 0.9357)
		Weighted median	33	0.0420	0.8281 (0.6905 − 0.9932)
		MR Egger	33	0.9095	1.0748 (0.3130 − 3.6905)
		RAPS	33	0.0966	0.8497 (0.7012 − 1.0297)

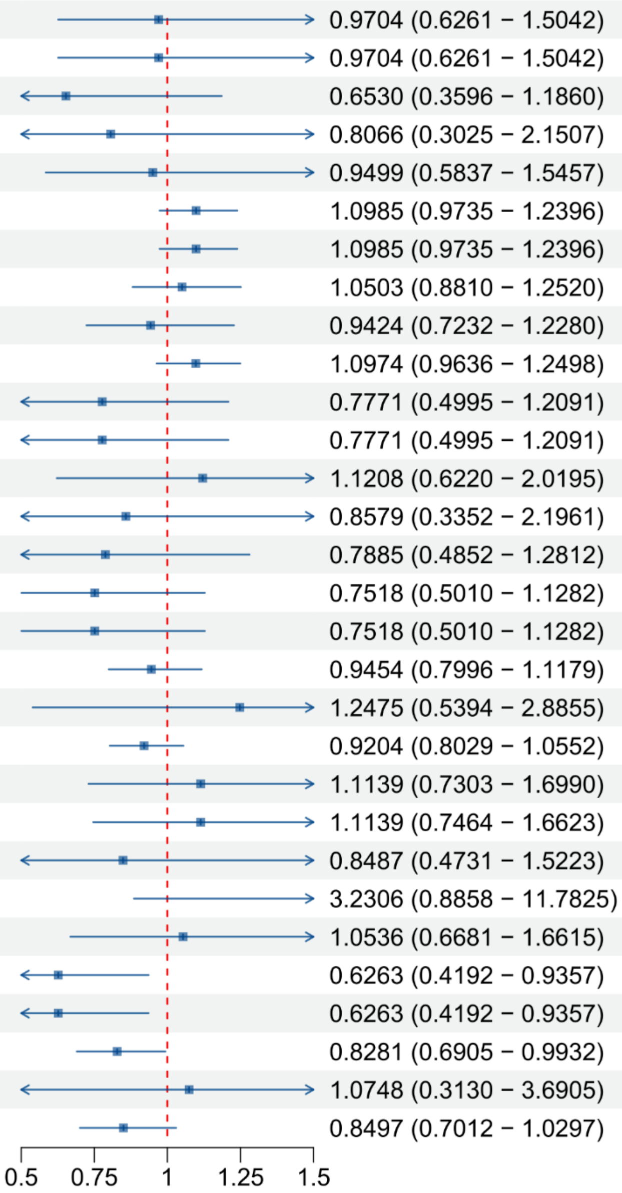

D.

Exposure	Outcome	Method	n.SNP	P.value	OR (95% CI)
Practice of adding artificial sweetener to cereal	T1DM	IVW	30	0.7192	1.1997 (0.4446 − 3.2368)
		IVW−MRE	30	0.7192	1.1997 (0.4446 − 3.2368)
		Weighted median	30	0.9032	1.0818 (0.3047 − 3.8414)
		MR Egger	30	0.6712	1.5316 (0.2184 − 10.7392)
		RAPS	30	0.9019	0.9104 (0.2045 − 4.0522)
Practice of adding artificial sweetener to cereal	T2DM	IVW	30	0.3633	1.1373 (0.8618 − 1.5009)
		IVW−MRE	30	0.3633	1.1373 (0.8618 − 1.5009)
		Weighted median	30	0.6785	1.0759 (0.7613 − 1.5205)
		MR Egger	30	0.0251	1.8456 (1.1112 − 3.0655)
		RAPS	30	0.2109	1.2302 (0.8892 − 1.7019)
Practice of adding artificial sweetener to coffee	T1DM	IVW	25	0.9768	1.0074 (0.6116 − 1.6595)
		IVW−MRE	25	0.9768	1.0074 (0.6116 − 1.6595)
		Weighted median	25	0.4341	1.3464 (0.6390 − 2.8371)
		MR Egger	25	0.5185	1.3742 (0.5314 − 3.5535)
		RAPS	25	0.5282	1.2228 (0.6546 − 2.2842)
Practice of adding artificial sweetener to coffee	T2DM	IVW	25	0.1898	0.9117 (0.7941 − 1.0468)
		IVW−MRE	25	0.1806	0.9117 (0.7963 − 1.0438)
		Weighted median	25	0.7654	0.9708 (0.7989 − 1.1796)
		MR Egger	25	0.1766	0.8305 (0.6396 − 1.0783)
		RAPS	25	0.3488	0.9315 (0.8029 − 1.0806)
Practice of adding artificial sweetener to tea	T1DM	IVW	28	0.7061	0.8969 (0.5095 − 1.5788)
		IVW−MRE	28	0.7061	0.8969 (0.5095 − 1.5788)
		Weighted median	28	0.9484	1.0258 (0.4735 − 2.2226)
		MR Egger	28	0.3877	0.5667 (0.1597 − 2.0115)
		RAPS	28	0.8197	1.0680 (0.6065 − 1.8806)
Practice of adding artificial sweetener to tea	T2DM	IVW	28	0.8802	0.9847 (0.8058 − 1.2033)
		IVW−MRE	28	0.8802	0.9847 (0.8058 − 1.2033)
		Weighted median	28	0.9733	0.9963 (0.8040 − 1.2347)
		MR Egger	28	0.9836	1.0048 (0.6382 − 1.5822)
		RAPS	28	0.6665	0.9570 (0.7837 − 1.1687)
Low calorie drink intake	T1DM	IVW	28	0.0153	0.6875 (0.5078 − 0.9307)
		IVW−MRE	28	0.0004	0.6875 (0.5588 − 0.8457)
		Weighted median	28	0.0932	0.6978 (0.4584 − 1.0622)
		MR Egger	28	0.1063	0.6091 (0.3407 − 1.0888)
		RAPS	28	0.0259	0.6841 (0.4899 − 0.9554)
Low calorie drink intake	T2DM	IVW	28	0.4564	0.9586 (0.8577 − 1.0714)
		IVW−MRE	28	0.4564	0.9586 (0.8577 − 1.0714)
		Weighted median	28	0.5715	0.9601 (0.8338 − 1.1055)
		MR Egger	28	0.5952	0.9421 (0.7580 − 1.1709)
		RAPS	28	0.5912	0.9666 (0.8539 − 1.0942)

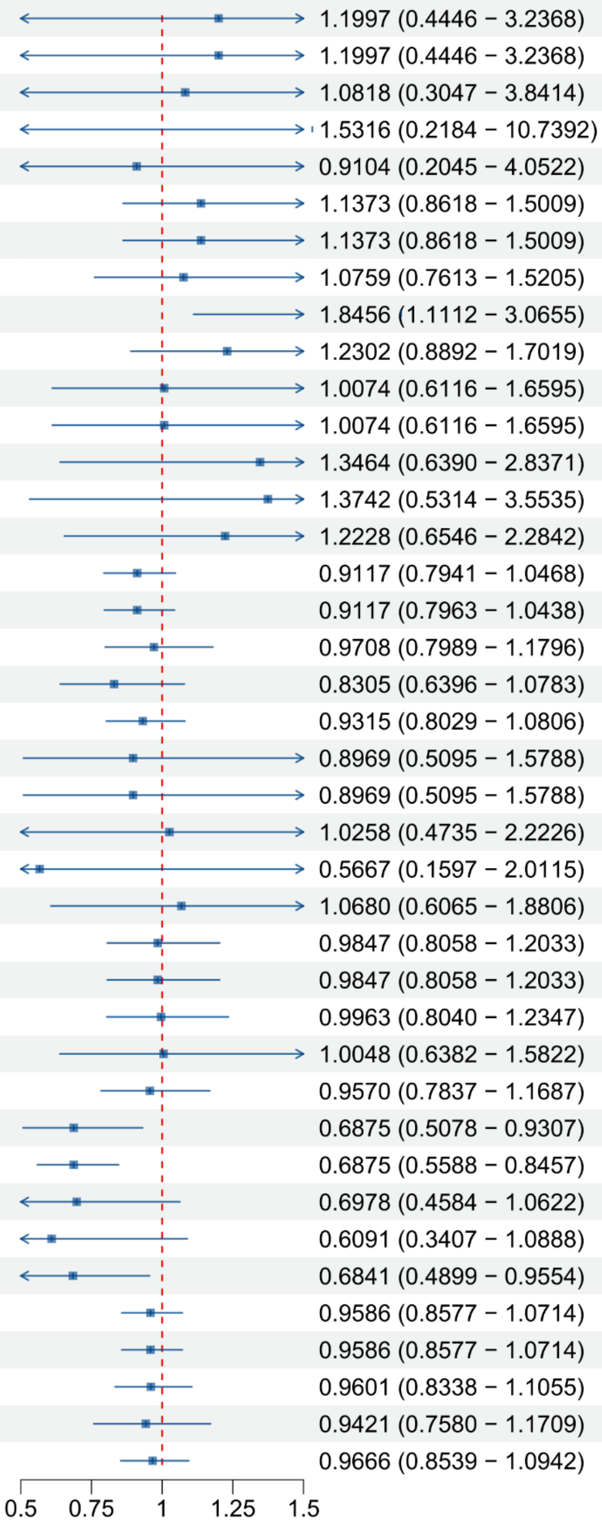

E.

Exposure	Outcome	Method	n.SNP	P.value		OR (95% CI)
Fruit liking	T1DM	IVW	9	0.7591		1.0903 (0.6274 − 1.8948)
		IVW−MRE	9	0.7569		1.0903 (0.6307 − 1.8847)
		Weighted median	9	0.5043		1.2888 (0.6119 − 2.7145)
		MR Egger	9	0.6516		1.9172 (0.1282 − 28.6797)
		RAPS	9	0.5538		1.1917 (0.6669 − 2.1294)
Fruit liking	T2DM	IVW	9	0.0988		0.7281 (0.4996 − 1.0613)
		IVW−MRE	9	0.0988		0.7281 (0.4996 − 1.0613)
		Weighted median	9	0.1748		0.8505 (0.6731 − 1.0746)
		MR Egger	9	0.9262		1.0950 (0.1717 − 6.9837)
		RAPS	9	0.2376		0.8107 (0.5722 − 1.1485)
Apple intake	T1DM	IVW	26	0.9858		0.9970 (0.7195 − 1.3817)
		IVW−MRE	26	0.9858		0.9970 (0.7195 − 1.3817)
		Weighted median	26	0.3614		0.8055 (0.5063 − 1.2816)
		MR Egger	26	0.9004		0.9406 (0.3637 − 2.4321)
		RAPS	26	0.8396		0.9617 (0.6590 − 1.4034)
Apple intake	T2DM	IVW	26	0.8010		1.0128 (0.9175 − 1.1179)
		IVW−MRE	26	0.8010		1.0128 (0.9175 − 1.1179)
		Weighted median	26	0.4811		0.9531 (0.8339 − 1.0894)
		MR Egger	26	0.7130		0.9473 (0.7122 − 1.2599)
		RAPS	26	0.9783		0.9985 (0.8973 − 1.1111)
Banana intake	T1DM	IVW	25	0.0670		0.7427 (0.5403 − 1.0210)
		IVW−MRE	25	0.0670		0.7427 (0.5403 − 1.0210)
		Weighted median	25	0.1214		0.6954 (0.4391 − 1.1012)
		MR Egger	25	0.7960		0.8952 (0.3905 − 2.0525)
		RAPS	25	0.1149		0.7295 (0.4928 − 1.0798)
Banana intake	T2DM	IVW	25	0.2400		1.0783 (0.9509 − 1.2228)
		IVW−MRE	25	0.2400		1.0783 (0.9509 − 1.2228)
		Weighted median	25	0.5524		1.0472 (0.8993 − 1.2195)
		MR Egger	25	0.1725		1.2611 (0.9131 − 1.7418)
		RAPS	25	0.0852		1.1205 (0.9843 − 1.2755)
Cherry intake	T1DM	IVW	27	0.7579		0.8591 (0.3270 − 2.2570)
		IVW−MRE	27	0.7536		0.8591 (0.3327 − 2.2181)
		Weighted median	27	0.9397		0.9464 (0.2271 − 3.9444)
		MR Egger	27	0.7807		1.2942 (0.2148 − 7.7963)
		RAPS	27	0.9485		0.9662 (0.3401 − 2.7449)
Cherry intake	T2DM	IVW	27	0.2635		0.8243 (0.5875 − 1.1566)
		IVW−MRE	27	0.2635		0.8243 (0.5875 − 1.1566)
		Weighted median	27	0.6287		0.8982 (0.5812 − 1.3880)
		MR Egger	27	0.1423		1.5536 (0.8786 − 2.7470)
		RAPS	27	0.9376		0.9819 (0.6223 − 1.5493)
Mango intake	T1DM	IVW	43	0.4501		1.4486 (0.5537 − 3.7896)
		IVW−MRE	43	0.4501		1.4486 (0.5537 − 3.7896)
		Weighted median	43	0.9103		0.9231 (0.2297 − 3.7107)
		MR Egger	43	0.2404		3.1270 (0.4791 − 20.4100)
		RAPS	43	0.4309		1.5019 (0.5459 − 4.1323)
Mango intake	T2DM	IVW	43	0.5964		1.0860 (0.8003 − 1.4737)
		IVW−MRE	43	0.5964		1.0860 (0.8003 − 1.4737)
		Weighted median	43	0.8079		1.0517 (0.7007 − 1.5784)
		MR Egger	43	0.7416		0.9039 (0.4978 − 1.6413)
		RAPS	43	0.8751		1.0288 (0.7222 − 1.4656)
Melon intake	T1DM	IVW	33	0.1419		1.7759 (0.8252 − 3.8219)
		IVW−MRE	33	0.1419		1.7759 (0.8252 − 3.8219)
		Weighted median	33	0.8569		1.1001 (0.3901 − 3.1028)
		MR Egger	33	0.6278		1.4201 (0.3489 − 5.7810)
		RAPS	33	0.5953		1.2951 (0.4987 − 3.3632)
Melon intake	T2DM	IVW	33	0.7268		1.0427 (0.8249 − 1.3179)
		IVW−MRE	33	0.7268		1.0427 (0.8249 − 1.3179)
		Weighted median	33	0.5949		1.0789 (0.8155 − 1.4272)
		MR Egger	33	0.7980		1.0583 (0.6884 − 1.6269)
		RAPS	33	0.6945		1.0433 (0.8444 − 1.2891)
Peach/Nectarine intake	T1DM	IVW	35	0.8430		1.0543 (0.6246 − 1.7797)
		IVW−MRE	35	0.7993		1.0543 (0.7013 − 1.5850)
		Weighted median	35	0.9259		0.9650 (0.4556 − 2.0439)
		MR Egger	35	0.1039		2.6044 (0.8484 − 7.9953)
		RAPS	35	0.8491		0.9467 (0.5387 − 1.6639)
Peach/Nectarine intake	T2DM	IVW	35	0.2740		1.1201 (0.9141 − 1.3725)
		IVW−MRE	35	0.2740		1.1201 (0.9141 − 1.3725)
		Weighted median	35	0.7498		1.0435 (0.8032 − 1.3557)
		MR Egger	35	0.8423		0.9561 (0.6162 − 1.4833)
		RAPS	35	0.3688		1.1289 (0.8666 − 1.4705)
Pineapple intake	T1DM	IVW	33	0.9749		1.0127 (0.4614 − 2.2226)
		IVW−MRE	33	0.9749		1.0127 (0.4614 − 2.2226)
		Weighted median	33	0.4125		1.5713 (0.5331 − 4.6308)
		MR Egger	33	0.3345		0.4568 (0.0954 − 2.1879)
		RAPS	33	0.7283		1.1579 (0.5063 − 2.6478)
Pineapple intake	T2DM	IVW	33	0.4265		0.9088 (0.7179 − 1.1504)
		IVW−MRE	33	0.4265		0.9088 (0.7179 − 1.1504)
		Weighted median	33	0.5286		0.9014 (0.6525 − 1.2450)
		MR Egger	33	0.7223		0.9159 (0.5667 − 1.4804)
		RAPS	33	0.4097		0.8971 (0.6930 − 1.1613)

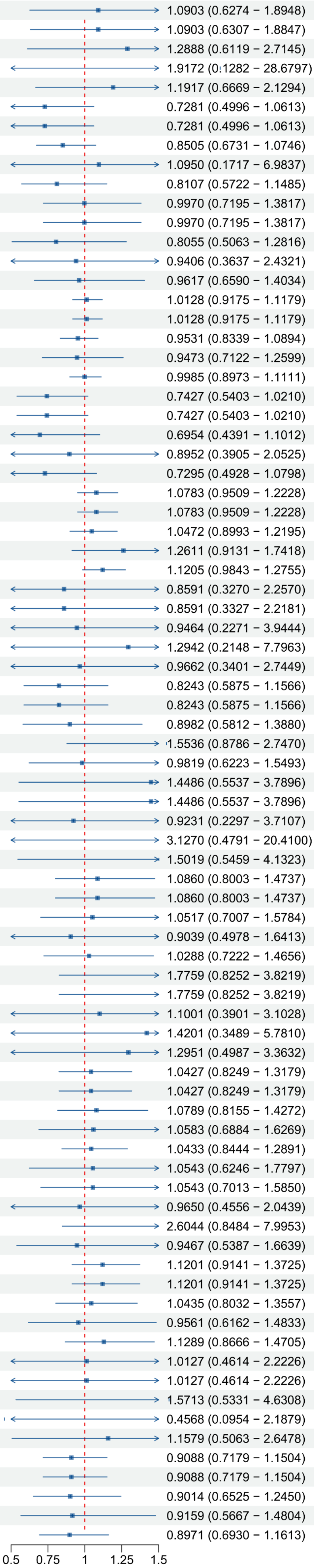

Supplement: Supplementary file 1 [file medi-104-e46479-s001.pdf]
